# A Flexible Fluid Delivery System for Rodent Behavior Experiments

**DOI:** 10.1523/ENEURO.0024-25.2025

**Published:** 2025-07-24

**Authors:** Bruno F. Cruz, Paulo Carriço, Luís Teixeira, Sofia Freitas, Filipe Mendes, Dario Bento, Artur Silva

**Affiliations:** ^1^Champalimaud Research, Champalimaud Foundation, Lisbon 1400-038, Portugal; ^2^NeuroGEARS Ltd, London NW1 7EA, United Kingdom

## Abstract

Experimental behavioral neuroscience relies on the ability to deliver precise amounts of liquid volumes to animal subjects. Among others, it allows the progressive shaping of behavior through successive, automated, reinforcement, thus allowing training in more demanding behavioral tasks and the manipulation of variables that underlie the decision-making process. Here we introduce a stepper motor-based, fully integrated, open-source solution, that allows the reproducible delivery of small (<1μl) liquid volumes. The system can be controlled via software using the Harp protocol (e.g., from Bonsai or Python interfaces), or directly through a low-level I/O interface. Both the control software and electronics are compatible with a wide variety of motor models and mechanical designs. However, we also provide schematics, and step-by-step assembly instructions, for the mechanical design used and characterized in this manuscript. We provide benchmarks of the full integrated system using a computer vision method capable of measuring across-trial delivery of small volumes, an important metric when having behavior experiments in mind. Finally, we provide experimental validation of our system by employing it in a psychophysics rodent task, and during electrophysiological recordings.

## Graphical abstract

**Figure:**
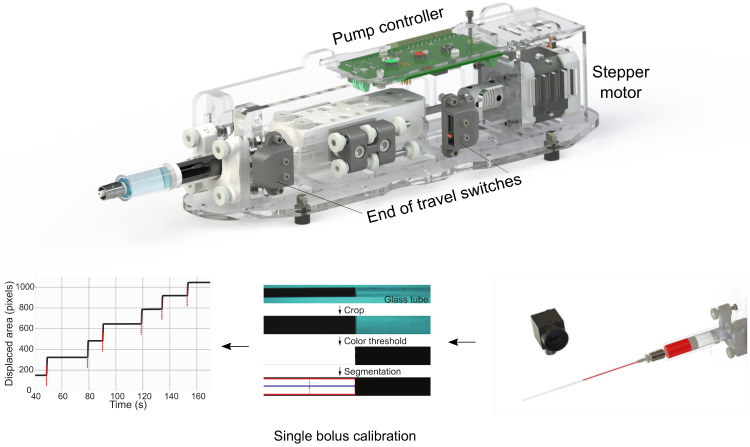
Visual Abstract

## Significance Statement

Liquid rewards are widely used in neuroscience behavior reinforcement. An open-source syringe pump designed for precise liquid delivery, while addressing the limitations of traditional gravity-based liquid delivery systems is presented. The new syringe pump system, built using off-the-shelf and 3D printed parts, offers liquid delivery with a microliter precision and it can be controlled via software or through a low-level I/O interface. The system’s performance is validated using a computer vision approach, and shown to be a suitable solution for rodent behavior and electrophysiological experiments.

## Introduction

Modern experimental neuroscience research relies on training animals in tasks that isolate or exacerbate demands for specific cognitive variables of interest (Gomez-Marin et al., 2014). Achieving stable performance in such paradigms typically involves the progressive reinforcement of simpler behaviors, a process known as “shaping” (Jones and Skinner, 1939). Among the various reinforcers used, liquid rewards have become a standard choice in many animal models due to their practicality and ease of use (Guo et al., 2014).

The most widely adopted liquid delivery systems in neuroscience are gravity-based systems, which use valves to control fluid flow. These systems determine the delivered volume based on the duration the valve remains open. While convenient and compact, gravity-based systems face several limitations. First, they are prone to calibration drift caused by changes in fluid resistance, such as biofilm buildup in tubing, necessitating frequent recalibration and maintenance. Second, the relationship between valve opening time and delivered volume is often non-linear, particularly for small volumes, requiring extensive calibration. Finally, these systems struggle to decouple the delivered liquid volume from the total delivery time, limiting their flexibility.

Active systems, such as syringe pumps, offer a promising alternative to address these challenges. Over the years, open-source syringe pump designs have been developed for diverse applications, including microfluidics (Lake et al., 2017; Booeshaghi et al., 2019; Park and Shin, 2024), chemistry (Cubberley and Hess, 2016; Garcia et al., 2018; Samokhin, 2020), and fluid delivery in behavioral experiments (Amarante et al., 2019). Commercially available syringe pumps are often prohibitively expensive, lack the flexibility required for diverse experimental needs, and are difficult to scale for high-throughput applications.

**Figure 1.**
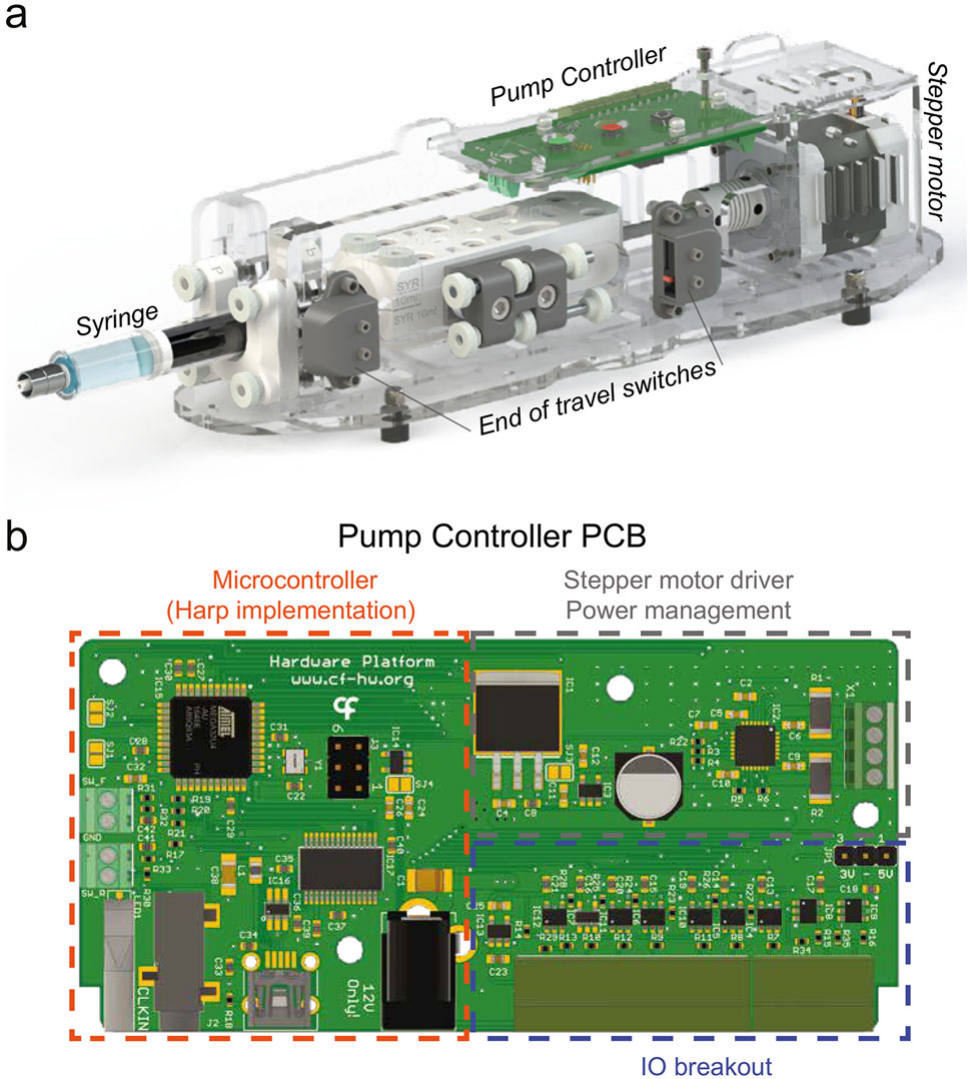
Syringe pump system. ***a***, 3D model of the fully assembled syringe pump system. Controller printed circuit board (PCB), syringe, switches, and stepper motor are highlighted. The assembly process is documented with step-by-step instructions, including detailed photographs. ***b***, Diagram of controller PCB. The three main sections of the board are highlighted: microcontroller, which implements the Harp protocol; motor driver and power, which provide the low-level logic to drive the stepper motor; and the I/O breakout, that affords users with input and output lines which can be used to control and monitor the function of the system, respectively. The modularity of the board design affords users the option to assemble a simpler version without the microcontroller block, for applications wherein low-level control is sufficient. This cheaper version is assembled using the same PCB schematic, with minimal changes to the board components, and can be found in an alternative bill of materials provided. See Materials and Methods for further details.

In this manuscript, we present and characterize an open-source syringe pump system designed specifically for neuroscience experiments, capable of reliably delivering liquid volumes in the microliter range (Fig. 1). The system incorporates the Harp protocol (Carvalho et al., 2024) and offers flexible control options to meet diverse experimental requirements. Integration with Bonsai (Lopes et al., 2015), a visual programming language widely used in neuroscience, further enhances the system’s capabilities by enabling real-time data acquisition, closed-loop tasks, and dynamic control of liquid delivery.

To evaluate the system’s performance, we conducted a thorough characterization of its fluid delivery accuracy and variability. Unlike traditional methods that measure average delivery volumes over multiple trials, we developed a computer vision-based assay to assess trial-to-trial variability in single-bolus events. This approach provides a more detailed understanding of the system’s performance, particularly for the small liquid volumes commonly used in rodent experiments.

We validated the syringe pump system in two experimental contexts. First, we demonstrated its ability to dynamically vary reward volumes in a rodent two-arm bandit task, showing that reward magnitude can quickly and reversibly influence choice behavior. Second, we confirmed the system’s compatibility with electrophysiological recordings, showing no detectable electrical artifacts during operation. These results highlight the system’s utility and reliability for a wide range of neuroscience applications.

## Materials and Methods

### Syringe pump construction

The presented syringe pump system is composed of a mechanical assembly, an electronic controller board, and an accompanying firmware/software stack that affords different levels of control over the system’s behavior. All resources (interface software, firmware, and mechanical designs) are available (Extended Data 1). These files are also freely available online from https://github.com/harp-tech/device.syringepump.

#### Mechanical construction

The mechanical assembly of the syringe pump was designed to be easily produced and assembled without any particular expertise. All parts necessary for the build are included in the bill of materials. Most parts are readily available off the shelf. The few custom parts can be laser-cut, 3d printed or sourced from third-party manufacturers, using the provided designs.

In the provided mechanical configuration, we opted for a NEMA 17 Stepper Motor (HT17-275 from Applied Motion) due to its high precision and torque rating. Specifically, the combination of motor step resolution (1.8 ∘/step) together with a 0.8 mm pitch length driving rod, leads to a theoretical linear resolution of 4 μm/step, which can be further increased by using step multipliers of up to 1/16. To characterize the system, we chose two glass syringes models with total volumes compatible with those routinely used for animal experiments (5 and 10 ml, 1,000 Series Syringes, Hamilton), leading to a single-full-step theoretical resolution of 0.33 and 0.66 μl/step. Nevertheless, the system should be able to be modified to use other syringes or stepper motors with little to no modifications to the provided designs.

#### Electronics

The syringe pump is controlled by a custom-made printed circuit board which is comprised of three main blocks: a microprocessor, an interface logic circuitry and, a motor driver (Fig. 1).

The microprocessor block consists of an ATxmega microprocessor (ATXMEGA128A4U-AU, Microchip Technology) that implements the Harp protocol (Carvalho et al., 2024), a USB to serial UART interface and a stereo phone jack for temporal synchronization between Harp devices. The Harp protocol is a lightweight binary messaging protocol that enables precise hardware-level timestamping, temporal synchronization with other Harp-compliant devices at acquisition time, easy logging and ingestion https://harp-tech.org/articles/python.html, and a standard serial communication protocol. Such standard allows the syringe pump to be flexibly controlled externally through the USB interface with any host implementing the Harp API [currently via Bonsai (Lopes et al., 2015) or Python https://github.com/harp-tech/pyharp].

The interface circuitry block consists of logic buffers that provide a direct low-level interface with the micro-stepper driver (A4988SETTR-T, Allegro MicroSystems) to the user (bypassing the microcontroller block). Moreover, it provides access to the I/O breakout that can be used to trigger protocol delivery with other external, transistor–transistor logic (TTL) compatible, devices, supporting input voltages up to 5 V.

The control system is compatible with other types of bipolar stepper motors with a maximum output drive capacity of 12 V and ±1.7 A.

#### Syringe pump user control

**Figure 2.**
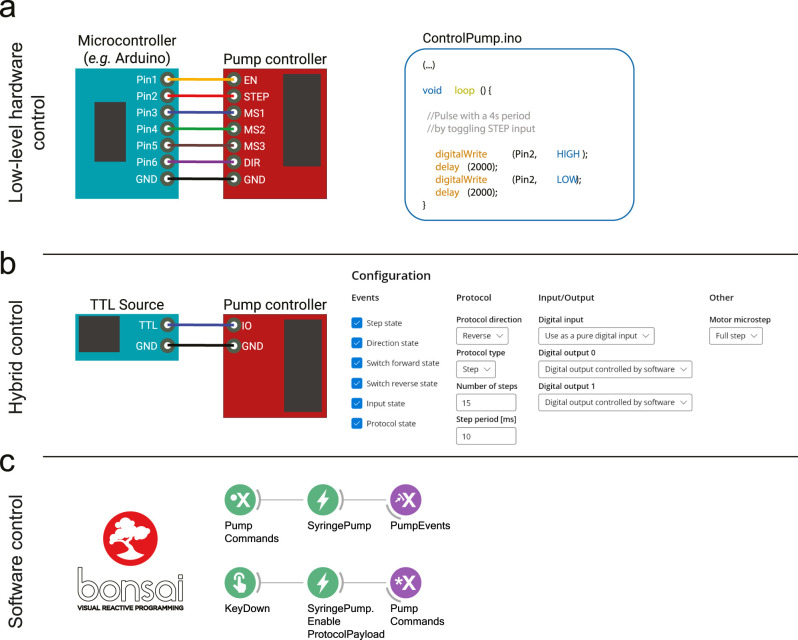
The current system affords three distinct levels of interface. ***a***, Low-level hardware control expects the user to fully define the control logic for the stepper motor driver. This can be achieved by implementing such logic in a microcontroller (e.g., Arduino, right textbox) that defines the state of all input control pins to the stepper motor driver. It should be noted that this mode does not require the full PCB to be populated, since it does not rely on the Harp core protocol implementation. ***b***, Trigger-based control allows the user to use an external trigger event (via transistor–transistor logic, TTL) to playback a pre-defined delivery protocol. The default protocol values (e.g., volume and flow rate) can be modified using a provided graphical-user interface, or Bonsai. ***c***, Software control allows the user to fully parameterize, and trigger, protocols from a computer host running Bonsai without the need for any external hardware triggers. Sub-millisecond synchronization can be achieved via Harp protocol, or by a configurable digital output event.

We designed the syringe pump system with three distinct interface levels that afford experimenters control over the pump according to their experimental needs. In addition to the software interface, which enables full control over all parameters of the syringe pump controller, two additional hardware interface levels are provided. These hardware interfaces facilitate the syringe pump integration into existing experimental setups running diverse software solutions (e.g., LabView, MATLAB) or other implementations that may not support or be easily upgraded to software-based solutions. This is particularly relevant for open-source microcontrollers (e.g., Arduino, Teensy) or proprietary controllers (e.g., Med Associates). In any of these cases, the pump control can be easily achieved by adding and configuring additional digital control lines to interface with the pump. In addition to Figure 2, we offer a brief description of the three available options:

- Low-level control—This option relies on directly controlling the I/O interface of the micro-stepper driver. This is achieved by using an external source (e.g., Arduino microcontroller) to generate the necessary input logic. Eight inputs lines are exposed: GND (ground), EN (enables the driver when low), MS1-3 (determines the step-size of the motor, 1 to 1/16 times a full-step), DIR (determines the direction of rotation), and STEP (triggers a micro-step). Additionally, the PCB exposes two outputs that correspond to “end-of-travel” switches, that can be used to implement operation safety-stops. This control option offers maximum flexibility without needing full board assembly but requires external logic control, which is often unnecessary and cumbersome for most uses.
- Trigger-based control—This option allows the user to configure a “protocol” that is triggered with the detection of a TTL rising edge. The protocol can be configured via a custom-designed graphic user interface (GUI; Fig. 2). Available settings currently include an option to run in “Step Mode,” wherein the user sets number, duration, and frequency of the steps per protocol. The behavior of the I/O pins can be configured via this GUI to provide users with hardware events that can be used to interface with third-party devices. It should be noted that, contrary to the previous mode, “end-of-travel” events (i.e., when the limit switches are triggered) are automatically handled by the controller, to prevent accidental mechanical damage to the system.
- Software control—The third option allows the user to control the syringe pump system through a software API. This option can be seen as an extension of the last, with the protocol being triggered by a software, instead of an hardware event. This option is available to any host running the Harp API, namely Bonsai. Bonsai is a visual programming language that specializes in asynchronous data acquisition and has seen growing adoption from the neuroscience community. Users can change an large number of settings (identical to the “triggered control” version) and trigger a protocol on any Bonsai event. As an example, the user can periodically deliver a constant amount of reward (Fig. 2), dynamically change the amount of liquid delivered on every trial, contingent on some behavior/physiological event, or trigger the delivery of water aligned to when the animal enters a given area of an arena. Finally, the Harp protocol allows the user access to hardware timestamped events (e.g., protocol onset), allowing for easy event alignment.

### Calibration

#### Large volume calibration

The pump is compatible with a wide variety of syringes. However, it is advisable to calibrate the full system to account for potentially significant differences across parts. Small volumes of delivered liquid are technically challenging to measure, as a result, calibrating liquid delivery systems is usually performed by repeating the same protocol a large number of times. This strategy rests on the assumption that single steps are relatively reproducible and the mean across several dozens to hundreds of repetitions is thus representative. We calibrated the system by varying the number of steps per protocol, which was then repeated 200 times, waiting 250 ms between the end of each protocol and the start of the next. We then weighted the amount of collected liquid to yield the total collected volume (we assumed a water density of 1 g/ml at room temperature). Single-protocol volume was thus given by Equation 1:

$$V_{\text{single}}(\mathrm{ml})=\frac{\text{Weight}(g)}{1(g/\mathrm{ml})}\;*\;\frac{1}{200},$$

To assess reproducibility across runs, we repeated this protocol 20 times. Depending on the experiment, we also adjusted the range of calibration values. Critically, all tested volumes laid on top of a linear calibration regime, and outcomes were consistent with the predicted theoretical volumes (Eq. 1).

We followed protocol identical to the syringe pump system to calibrate the solenoid valves (LHDA1231215H, Lee). Briefly, we calibrated the valves by setting the water reservoir (a 20 ml plastic syringe) at a height identical to what we routinely use in our laboratory for behavioral experiments (∼30 cm). We systematically varied the opening time (between 10 and 150 ms) to yield different delivered volumes. Both the number and time between trials were identical to the ones used in the pump systems. Hardware control logic was implemented in an Arduino Microcontroller (Arduino Mega, Arduino).

#### Single-bolus calibration

**Figure 3.**
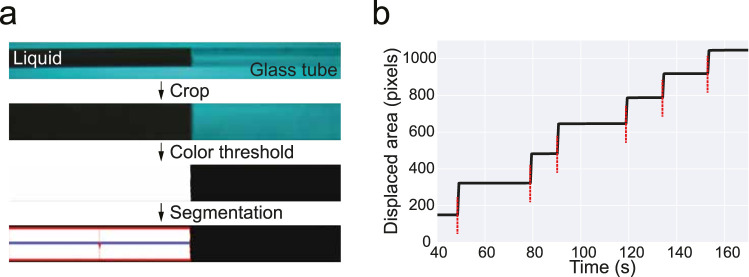
Tracking small single-bolus events. ***a***, Schematic of the computer vision algorithm for measuring small microliter range volumes. From top to bottom: cropped, thresholded, and segmented the area of the capillary filled with liquid. Area was taken as a proxy of delivered volume (see Materials and Methods for further details). ***b***, Example trace of the measured area in ***a***, as a function of time during one of the experiments. Red vertical dashed lines represent liquid delivery events.

To characterized the delivery of single small volume events we followed the following protocol. The end of a syringe was fitted with a plastic adapter with a small glass capillary (Model 211713, Vitrex) glued to the end. A computer vision camera (Model FL3-U3-13S2C-CS, FLIR) was used to image the capillary. In order to increase the contrast between liquid and background, we diluted a small amount of red colorant (Erythrosin B, Sigma-Aldrich) that, when combined with LED illumination, resulted in the image shown in Figure 3. We note that coloring was only added for benchmarking single-bolus calibration.

Video acquisition and processing were implemented in Bonsai. To measure the amount of displaced liquid inside the capillary, we defined a region of interest, and binarized the image by thresholding the pixel values in RGB color space. We used this binarized image to segment the area corresponding to the displaced liquid volume. We choose the horizontal axis of the region as our reported metric. Data were acquired at 10 frames-per-second and synchronization was achieved by sending a short TTL pulse to the camera each time a protocol was started. Syringe pump control logic was implemented using the low-level control mode of the system and an Arduino microcontroller.

Unless otherwise stated, the inter-pulse-interval was set to 4 ms. Before each run, up to 5 small pulses were applied to ensure that every protocol started from comparable conditions. These pulses were discarded from all analyses. The interval between each protocol was drawn from a uniform distribution (10–20 s). Due to capillary size, larger volumes required cleaning the tube between trials. We cleaned it by flushing ethanol and air-drying it.

For the dynamic flow rate experiment shown in Figure 5, the inter-pulse-interval was systematically varied to control the output flow rate. We estimate flow rate by linearly fitting, on each trial, the displaced area to time.

### Experimental validation

#### Animal behavior experiments

Two Long Evans, 5 month-old, rats were trained on a Two-armed bandits task (Ito and Doya, 2009). Briefly, subjects were placed in an experimental box with access to four nose ports, two for initiation, and two for reward delivery. Reward volumes were drawn from a pre-defined set of calibrated values (3.46, 6.92, 13.84, 27.68, and 55.36 μl) and delivered using the syringe pump system. A light on top of the initiation ports signaled trial availability. After initiating, animals were allowed to collect a reward at one of the two side-reward ports. The amount of water delivered at any port was constant within a given session block, but always different across the two ports. After reaching a preference criteria of >80% toward the highest-reward nose-port over the last 15 trials, a block transition would be triggered, the trial initiation switched to the opposite nose-port (e.g., odd and even numbered blocks would be initiated at the north and south nose ports, respectively). At block transitions, we forced reward amounts on each nose-port to be different, but otherwise allowed for any other combination. In particular, the highest rewarded nose-port need not to change between blocks. Finally, we report the noise levels produced by the system during operation (in dB): room noise level (pump off) = 50, inside box and 50 cm away from the pump = 61, 20 cm away from the pump = 69, 5 cm away from the pump = 82.

#### Compatibility with electrophysiological recordings

Previous studies report induced electrical artifacts from some syringe pump systems (Amarante et al., 2019). We thus tested the compatibility of our system with electrophysiological techniques by recording these signals while triggering the micro-stepper driver in close proximity. In short, mice (8–10 weeks old) were anesthetized with Isoflurane (1–2% at 0.8 L/min) and a small metallic head-post was cemented to the skull. Animals were given a dose of Carprofen on the day of the surgery, and were allowed to recover for a minimum of 5 days. Prior to the recording session, a circular craniotomy (1.5 mm of diameter) was opened above the dorsal striatum. During acquisition, animals were head-restrained by a custom-made apparatus and allowed to run on top of a rotatable cylinder. A silicon probe (ASSY 77-H2, Cambridge NeuroTech) was slowly lowered (2–10 μm/s) into the dorsal striatum (∼3 mm from brain surface). Headstage data was digitized at 30 kHz and acquired using the Open-Ephys acquisition board and Bonsai. In order to align electrophysiology data with pump events, we used the aforementioned “triggered-based control” mode and configured one of the controller’s outputs to report STEP events via TTL, which was then acquired with the same Open-Ephys acquisition board. The syringe pump was positioned 20 cm away from the animal without any electromagnetic shielding material in-between. Protocols were manually triggered by the experimenter. Electrophysiological data shown in Figure 9 was minimally processed by applying a Butterworth digital bandpass filter (0.3–8 kHz). All animal care and experimental procedures were carried out according to the European Directive 2010/63 and pre-approved by the competent authorities, namely, the Champalimaud Foundation animal care committee’s regulations.

## Results

### Characterization of sub-microliter volume delivery

We first focused on benchmarking the delivery of small liquid volumes. Such events are experimentally challenging to measure accurately. Other systems have relied on inferring the average volume of a single-bolus, by averaging repeated measurements. Despite straightforward, such method cannot resolve trial-to-trial variability. To circumvent this issue, we developed a quantitative computer vision-based assay, that can provide a metric of such variability (Fig. 3). Our methods relies on reliably segmenting the area occupied by water in a cross section of a small-diameter glass capillary with well-characterized and reproducible dimensions. Assuming Area_pixels_ ∝ Volume_ml_, we can use it as a linear proxy for delivered volume.

**Figure 4.**
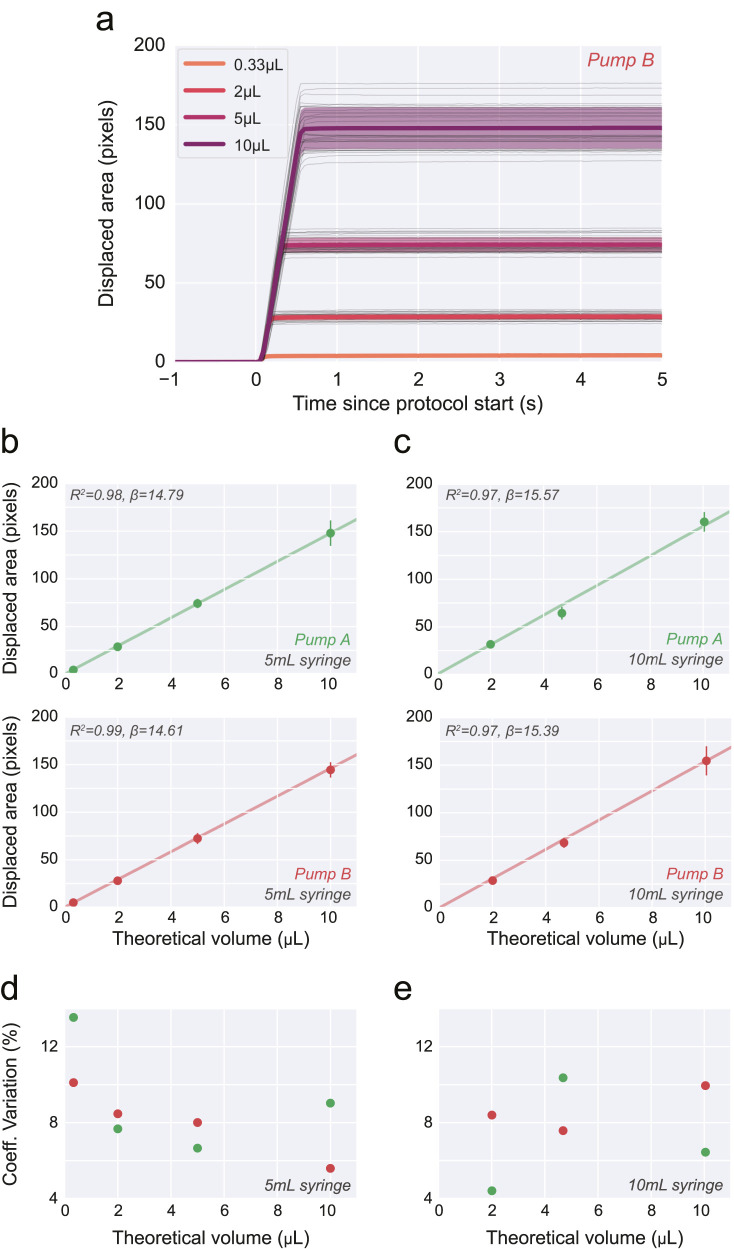
Single-bolus protocol calibration. ***a***, Time course of displaced area aligned on protocol onset (*t* = 0) for four distinct theoretically expected delivered volumes. Thin and thick lines correspond to single trials, and averages for a given expected volume, respectively. Shaded area depicts s.t.d. ***b, c***, Total displaced area for the four protocols. Each point shows mean ± s.t.d. across 30 replicates, per volume, in two different pumps (*Pump A* and *Pump B*, green and red, respectively) and using two different glass syringe sizes (5 and 10 ml, ***b*** and ***c***, respectively). ***d, e***, Coefficient of variation (s.t.d./mean) calculated from the data shown in ***b*** and ***c***, respectively.

Using this approach, we picked four distinct volumes, in a range relevant to rodent behavior experiments (0.33–10 μl). Based on the mechanical specifications of our system and mounted syringe models, we calculated the number of steps expected to achieve such volumes. Next, using the videography data, we constructed a “protocol-onset triggered” trace that can be used to quantify single-trial delivered volume, as well as its fluid kinetics (Fig. 4*a*). This procedure provides an estimate of variability across single delivery protocols, on top of revealing a close linear relationship between theoretically predicted and delivered volume. This result held true not only across different setups (*Pump A* vs *Pump B*), but also across mounted syringes with different dimensions (10 ml vs 5 ml; Fig. 4*b,c*). Finally, while the spread of single-trial delivered volumes grew with theoretically calculated values, its coefficient of variation remained relatively stable in all conditions (Fig. 4*d,e*).

### Flow rate modulation

**Figure 5.**
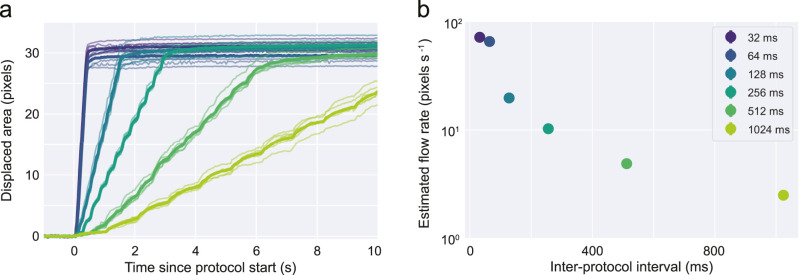
Syringe pump affords dynamic control over the flow rate. ***a***, Time course of delivered volume (displaced pixel area) aligned on protocol onset (*t* = 0) for different inter-protocol-interval values (IPI). Thin and thick lines represent single trials, and averages, for a given IPI, respectively. ***b***, Estimated flow rate (pixels s^−1^) for all tested IPI (mean ± s.t.d., *n* = 6 trials for each IPI).

Our characterization method also affords the chance of measuring single-trial time course dynamics (i.e., flow rate, pixels s^−1^). Theoretically, flow rate should be a function of the number of steps the driver takes per unit time. As a proof-of-concept, we performed an experiment where we varied the time between each train of pulses (protocol) and characterized how these changes affected the small amounts of liquid delivered per unit time (Fig. 5). As predicted, the displaced area in the capillary per unit time monotonically decreases with larger intervals between protocols. While this relationship is clearly not linear, the reproducibility of such dynamics across trials affords the chance of precisely calibrating the flow rate for a specific system.

### Calibration of large volumes

**Figure 6.**
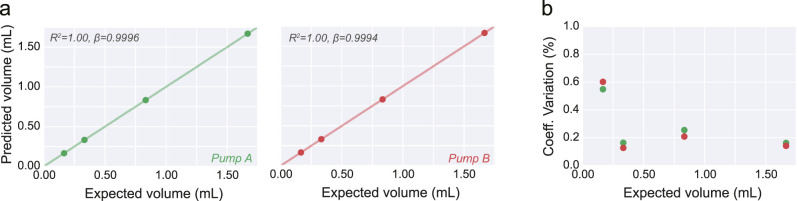
Large volume protocol calibration. ***a***, Delivered volume (mean±s.t.d.) across 20 performed delivery protocols for four distinct large volume amounts, in two independent pump systems (*Pump A* and *Pump B*, green and red, respectively). “Predicted volume” was calculated by weighting the delivered liquid and assuming a water density of 1 g/ml. “Expected volume” was calculated using the theoretically displaced syringe volume. *R*^2^ and slope (*β*) resulting from the linear fit to the data are shown in the top-left corner. ***b***, Coefficient of variation (s.t.d./mean) calculated from the data shown in ***a***.

After characterizing the small-bolus delivery capabilities of the system, we decided to follow a typical, and more experimentally amenable, calibration protocol. Typically, experimenters rely on measuring the outcome of several hundred repeats of the same protocol. While this strategy effectively blinds the researcher to inter-protocol variability, it allows the measurement of larger volumes, with smaller systematic errors, and thus the estimation of an average single-protocol delivery volume. Following such a protocol (see Methods), we demonstrate how calibration curves can be obtained from the setup, as well as validate the theoretically predicted amount of delivered volume calculated from the physical specifications of the system (Fig. 6). As predicted, both the quality (*R*^2^), and the slope (*β*), of the linear fit highlight the high-quality match between expected and measured volumes (Fig. 6*a*). Despite not being stable throughout the full range of probed delivered volumes, the coefficient of variation that results from measurements of this method is strikingly low (Fig. 6*b*).

### Calibration stability and comparison against gravity-based systems

**Figure 7.**
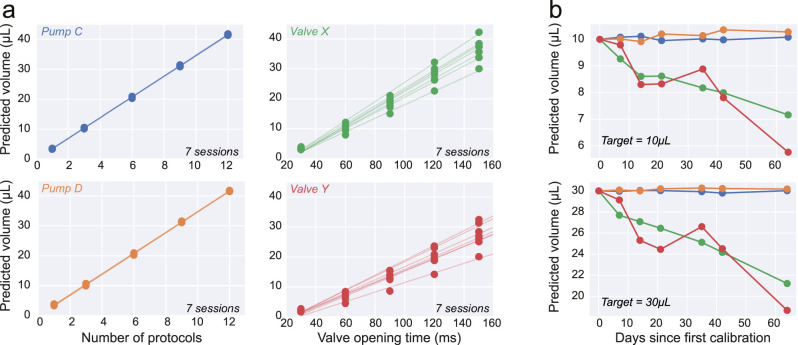
Comparison of calibration stability between the gravity-based solution and the presented pump system. ***a***, For each system, the calibration (see Materials and Methods) consisted of measurements of delivered liquid as a function of the number of pre-programmed delivery protocols, or valve opening time, for the *Pump* (left) and *Valve* (right) systems, respectively. Individual fits correspond to a single-day calibration protocol. For each system, we tested two devices independently (distinct colors). ***b***, Predicted delivery volume for two arbitrary volumes (10 and 30 μl, top and bottom, respectively) based on the calibration linear fits derived from ***a***. For each day, the fit calculated from day 0 was used as to infer that value would have been delivered had the calibration curve remained the same.

In the previous section, we show how calibration of the system can be achieved in an experimentally amenable fashion. Whilst such a procedure should take no more than a few minutes, it can quickly become unfeasible if several water delivery systems are routinely used simultaneously. As a result, we next benchmarked how stable the calibration is by performing longitudinal, weekly, calibration procedures. Our data shows that, at least, up to one month after the initial procedure (*Day 0*), calibration curves are identical (Fig. 7*a*, left).

Next, we considered the most common alternative to a syringe pump system, in experimental behavior neuroscience: gravity-based systems. These systems rely on a liquid reservoir at a higher potential than the system’s outlet. While simpler and generally more affordable, these systems are usually subject to changes in the fluid path resistance that lead to systematic differences in delivered volume over time. This problem can be partially circumvented by investing time on regular setup calibration and maintenance, which tends to hinder the scalability of this solution.

To compare the stability of the presented syringe pump system versus a common gravity-based system, we decided to perform and track regular calibration results of both the systems (Fig. 7).

A calibration procedure identical to the one described in the last section was followed. As expected, both systems show daily calibration curves consistent with a linear behavior (Fig. 7*a*). However a clear change in slope over days can be observed in both valves (Fig. 7*a*). Using the daily calibration values, one can ask: “How much liquid volume would be delivered, had the experimenter not performed the calibration procedure, and kept using the values from Day 0” (Fig. 7*b*). This analysis revealed that gravity-based valve systems steadily drift over days, whereas syringe pumps remained stable. The general decrease in delivered volume is likely due to a drop in flow rate due to the accumulation of biofilm in the tube and valves.

### Experimental use cases

We next show two examples of the presented syringe pump system use in a neuroscience laboratory setting. Example experiments highlight both the day-to-day easy of operation and also the compatibility of the system with other methods.

**Figure 8.**
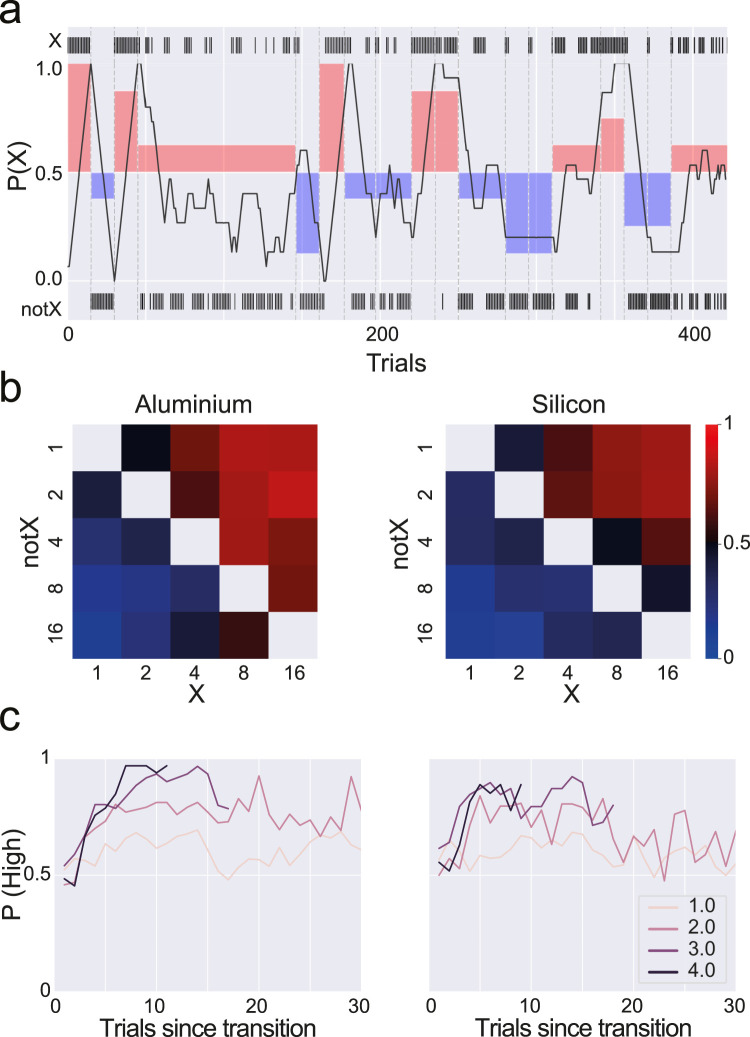
Rats quickly reverse choice preference on delivered liquid reward amount. ***a***, Example session. Animals alternate between the two available choices (“X” and “notX,” respectively) in a way that appears consistent with the highest-value choice (shaded area). Thick dark line depicts the probability of choosing “X” in a rolling window of 15 trials. ***b***, Probability of choosing reward giving nose-port X as a function of the possible reward amount combinations given by each nose-port. Values in the heatmap axes are the number of protocols given on each reward delivery, by each reward nose-port (X and notX). ***c***, Probability of choosing the highest rewarded side over the trials following block transition. Lines correspond to the absolute value of the difference between rewards at each nose-port in logarithmic units (base 2). The highest the difference between the two rewards available on a particular block, the sooner animals converge to the highest rewarded nose-port, suggesting the need for fewer reward samples (trials) as the difference between rewards increases.

In a first example, we tested rats in a two-arm bandit task, where delivered reward volume was parametrically varied. On each block (see Materials and Methods), the amount of reward at each option was randomly drawn from a set of five possible values. Both tested animals (*N* = 2) appeared to be sensitive to the relative amount of reward delivered and showed a systematic preference toward the highest-reward amount option, in a manner consistent with its relative difference in magnitude (Fig. 8). This preference quickly reversed at block transition, suggesting that animals did not need to rely on accumulating across many trials to determine the highest-value arm.

**Figure 9.**
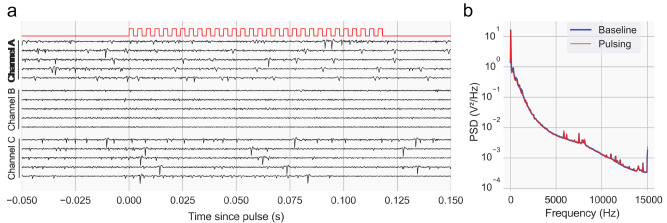
Compatibility with electrophysiological recordings. ***a***, Example single-trial traces (four trials) for three simultaneously recorded channels (A–C) aligned to the start of a protocol of several pulses (red trace). See Materials and Methods for further details. Notice the presence of spiking activity in Channels A and B, along with the lack of any clearly identified stepper motor-induced electrical artifact. ***b***, Correspondent average power spectral density plot for all channels in the recorded session (*n* = 32). Baseline and Pulsing correspond to a period before and during the syringe pump controller step trains, respectively.

In parallel, we also tested the system’s compatibility with electrophysiological recordings, a widespread technique in the field known to be susceptible to electromagnetic interference (Amarante et al., 2019). We performed acute recordings from a mouse’s brain (see Materials and Methods) while simultaneously operating the syringe pump (Fig. 9). We leveraged one of the digital outputs in the board to send a pulse on each “STEP” instruction, thus providing precise sub-millisecond alignment to the activity traces. Our data showed a clear absence of artifacts aligned to, or surrounding, each pulse, while still preserving easily identifiable single-unit spiking activity. Moreover, power spectral density analysis of epochs during and around the system’s operation failed to reveal differences between the two epochs. We conclude that the system does not introduce any detectable electrical artifacts, and thus can be used in conjunction with other electrophysiological recording systems.

## Discussion

Inspired by previous projects (Wijnen et al., 2014; Amarante et al., 2019), we present a fully integrated, and characterized system for microliter range fluid delivery targeted toward neuroscience behavior experiments.

We provide an alternative to gravity-based passive systems, by designing a scalable and affordable syringe pump system solution that allows for dynamic control over reward delivery, without suffering from the long-term stability issues often associated with the former systems. Additionally, we also highlight the afforded control gained over flow rate that such an non-passive system allows.

Nevertheless, limitations of the current system should be taken into consideration. First, compared to simple gravity-based solutions, our system has a significant larger spatial footprint and requires a larger upfront investment. These should be taken into consideration, especially for large-scale or resource constrained projects. Second, while it is technically feasible to automate refilling using external valves, the process remains more complex than the straightforward refill procedures of traditional gravity-fed systems. Third, the current mechanical design effectively caps the maximum size to 12 ml syringes.

In addition to the mechanical design of the syringe pump, we also developed a custom PCB that not only controls the presented pump but can also serve as a generic controller for various types of stepper motor-based syringe pumps. Since all the design files for the system are freely available, users can design and assemble their own pump configurations. Furthermore, by implementing the Harp protocol, the firmware affords precise timestamping and synchronization with other Harp devices, facilitating scalable and reliable experimental data collection pipelines.

To ensure seamless integration with already-existing experimental rigs (such as replacing gravity-based fluid delivery systems), we offer users with several options to interface with the system, affording a very flexible configuration and compatibility. By using pre-existent controllers available in the experimental setups, the syringe pump can be fully controlled through its low-level interface, a trigger-based protocol, the GUI or the Harp API. The integration of this API is already available in Bonsai, which is particularly relevant since it leverages the capabilities of the syringe pump, by offering a powerful tool for real-time data stream processing, video acquisition, close-loop tasks and real-time data visualization, in a visual programming language environment.

We also present a simple method which allowed us to provide an extensive characterization of trial-to-trial variability in the fluid delivery dynamics, an important stack of tests when considering the intended use of this system for rodent behavior experiments. Traditionally, fluid volume characterization relies on repeating multiple fluid deliveries and then weighing the total volume. However, this approach only provides the mean volume over multiple deliveries and fails to assess variability across single fluid delivery events. As we show in our results, the volume of these events is variable. Such variability is likely the result of factors such as defective rods, material hysteresis, syringe construction and measurement methods. Given how difficult it may be to completely remove some of these sources of variability, it highlights how important it is to characterize fluid delivery systems at this resolution.

Importantly, we validated the system under common neuroscience use cases by deploying it during rodent behavioral tasks, where we demonstrated systematic changes in choice preference based on delivered reward amount. Although this study focused on water, the same system has since been used with other solutions (e.g., sucralose; Silva et al., 2024). Finally, electrophysiological recordings confirmed that the system does not introduce electrical artifacts.

10.1523/ENEURO.0024-25.2025.d1 Data 1 Resource files for the interface software, firmware, mechanical designs and assembly. Download Data 1, ZIP file.

10.1523/ENEURO.0024-25.2025.d2 Data 2 Video highlighting the syringe pump assembly, liquid handling and delivery using the provided GUI. Download Multimedia/Extended Data, ZIP file.

## Synthesis

Reviewing Editor: Mark Laubach, American University Decisions are customarily a result of the Reviewing Editor and the peer reviewers coming together and discussing their recommendations until a consensus is reached. When revisions are invited, a fact-based synthesis statement explaining their decision and outlining what is needed to prepare a revision will be listed below. The following reviewer(s) agreed to reveal their identity: NONE.

Your paper was reviewed by two experts and their comments are given below in full. Please review your paper to address all points that were raised.

From conversation about your paper in our discussion forum, it was suggested that you consider including a video showing the use of the device. This can help motivate other labs to use your tool.

Reviewer 1

In this manuscript, the authors design a flexible syringe pump system that precisely measures the volume delivery on each trial. This feature is valuable, and the equipment will be useful for the system neuroscience field. I like the syringe, but I have some major concerns that need to be addressed to improve the manuscript.

1) Regarding pump calibration, is the calibration different for different solutions to water? For example, instead of water, some experimenters studying devaluation used sucrose.

2) Regarding the added color, it is not clear if this color is in contact with the drinking water and if it is, it changes the taste of water.

3) How reliable is this equipment for 24 hr recordings or at least is not specified for how long they can continuously use it. In this regard they compare the system versus gravity base valves. However, it is not compared nor cited against individual air-pressure system valves currently used for 24 hrs in psychophysical tasks in rats in equipment they called homegustometer. See their Figure 11 https://doi.org/10.1371/journal.pone.0300544

4) They did not measure the dB the stepper motor produced is this sound affecting the animal behavior?

5) Figure 9 presents evidence that activation of the stepper motor is compatible with electrophysiological recordings. However, it is not shown if the equipment is compatible with a contact lickometer or with a photobeam sensor that can synchronize the delivery of each drop with a single lick or simply deliver the entire volume and then the mouse collected with many licks? It is well known that contact lickometers and sometimes photobeams induce electrical artifacts not the stepper motors.

6) It will be illustrative to show a video of a rat and/or mouse drinking.

7) How sure are the authors that the mouse is drinking the entire volume delivered or that some volume is dropping out of the mouth? A video recording will help to clarify this point.

8) Did the authors show psychophysics in rats but not in mice? Did authors try to do psychophysics on mice, too? Is this syringe good enough to induce learning in mice with a psychophysical task on mice?

9) The conclusion is missing a section of limitations and caveats.

Reviewer 2

This study presents an open-source syringe pump designed for precise liquid delivery and addresses the limitations of traditional gravity-based liquid delivery systems.

1) In the introduction, it is necessary to make general corrections to the language.

2) Line 43: Revise 'Open-source solutions based on syringe pump designs have been developed over the years to target microfluid'

3) Lines 9 and 93: Correct the usage of e.g.

4) Briefly introduce the Harp Protocol and its benefits regarding what is often available.

5) Line 107: What would be the benefit of using Low-level control and Trigger Based Control user control over the Software Control. What are the possible gains for the user? If there aren't, why keep them?

6) Line 195: Was the electrophysiological recording made with the same two mice described in the previous topic?

7) Line 186: Why were only four values selected for validation, and what influenced the choice of these values? How do you justify that this amount of value is enough for validation?

8) Figure 4: What could explain the diverse coefficient of variation?

9) Figure 9: To see if the system could cause noise, I expected information regarding the characterization of the expected noise (frequency, amplitude), and the authors show without filtering that this specific artifact is not present in this application. The Butterworth filtering could have masked the presence of this noise.

## References

[B1] Amarante LM, Newport J, Mitchell M, Wilson J, Laubach M (2019) An open source syringe pump controller for fluid delivery of multiple volumes. eNeuro 6:ENEURO.0240–19.2019. ISSN 2373-2822. 10.1523/ENEURO.0240-19.2019 PMC6734045 31416819

[B2] Booeshaghi AS, da Veiga Beltrame E, Bannon D, Gehring J, Pachter L (2019) Principles of open source bioinstrumentation applied to the poseidon syringe pump system. Sci Rep 9:12385. ISSN 2045-2322. 10.1038/s41598-019-48815-9 31455877 PMC6711986

[B3] Carvalho F, Silva A, Cruz B, Frazao J, Lopes G (2024) Harp: a standard for reactive and self-synchronising hardware for behavioural research (v1.13.0). Zenodo 10.5281/zenodo.15874648.

[B4] Cubberley MS, Hess WA (2016) An inexpensive programmable dual-syringe pump for the chemistry laboratory. J Chem Educ 94:72–74. ISSN 1938-1328. 10.1021/acs.jchemed.6b00598

[B5] Garcia VE, Liu J, DeRisi JL (2018) Low-cost touchscreen driven programmable dual syringe pump for life science applications. HardwareX 4:e00027. ISSN 2468-0672. 10.1016/j.ohx.2018.e00027

[B6] Gomez-Marin A, Paton JJ, Kampff AR, Costa RM, Mainen ZF (2014) Big behavioral data: psychology, ethology and the foundations of neuroscience. Nat Neurosci 17:1455–1462. ISSN 1546-1726. https://www.nature.com/articles/nn.3812. https://doi.org/10.1038/nn.3812 25349912 10.1038/nn.3812

[B7] Guo ZV, et al. (2014) Procedures for behavioral experiments in head-fixed mice. PLoS One 9:e88678. ISSN 1932-6203. https://journals.plos.org/plosone/article?id=10.1371/journal.pone.0088678. https://doi.org/10.1371/journal.pone.0088678 24520413 10.1371/journal.pone.0088678 PMC3919818

[B8] Ito M, Doya K (2009) Validation of decision-making models and analysis of decision variables in the rat basal ganglia. J Neurosci 29:9861–9874. ISSN 0270-6474. 10.1523/JNEUROSCI.6157-08.2009 19657038 PMC6666589

[B9] Jones FN, Skinner BF (1939) The behavior of organisms: an experimental analysis. Am J Psychol 52:659. ISSN 00029556. https://www.jstor.org/stable/1416495?origin=crossref.https://doi.org/10.2307/1416495

[B10] Lake JR, Heyde KC, Ruder WC (2017) Low-cost feedback-controlled syringe pressure pumps for microfluidics applications. PLoS One 12:e0175089. ISSN 1932-6203. 10.1371/journal.pone.0175089 28369134 PMC5378403

[B11] Lopes G, et al. (2015) Bonsai: an event-based framework for processing and controlling data streams. Front Neuroinform 9:7. ISSN 1662-5196. 10.3389/fninf.2015.00007 25904861 PMC4389726

[B12] Park SB, Shin JH (2024) Open-source spring-driven syringe pump with 3D-printed components for microfluidic applications. HardwareX 19:e00550. ISSN 2468-0672. 10.1016/j.ohx.2024.e00550 39104615 PMC11299592

[B13] Samokhin AS (2020) Syringe pump created using 3D printing technology and Arduino platform. Fresenius J Anal Chem 75:416–421. ISSN 1608-3199. 10.1134/S1061934820030156

[B14] Silva A, Carriço P, Fernandes AB, Saraiva T, Oliveira-Maia AJ, Alves da Silva J (2024) High-precision optical fiber-based lickometer. eNeuro 11:ENEURO.0189-24.2024. https://www.eneuro.org/content/11/7/ENEURO.0189-24.2024. https://doi.org/10.1523/ENEURO.0189-24.2024 10.1523/ENEURO.0189-24.2024 PMC11258538 39025674

[B15] Wijnen B, Hunt EJ, Anzalone GC, Pearce JM (2014) Open-source syringe pump library. PLoS One 9:e107216. ISSN 1932-6203. 10.1371/journal.pone.0107216 25229451 PMC4167991

