# Supplementary material for: A Flexible Fluid Delivery System for Rodent Behavior Experiments
Source: eNeuro. 2025 Jul 24;12(7):ENEURO.0024-25.2025. doi: 10.1523/ENEURO.0024-25.2025 (PMC12320920; doi:10.1523/ENEURO.0024-25.2025)

**Assembly instructions** (some images were partially covered to avoid breaking anonymity)

**Note:** Exploded view with the identification number of each one of the parts is available in a separate document.

1. Laser cut the parts available in the repository. For the SHPSP_Frame_PC_V1_HAMILTON_SYR_10ml_drawing_GB_LR_5mm.pdf file a 5mm acrylic sheet must be used, while for the SHPSP_Frame_PC_V1_HAMILTON_SYR_10ml_drawing_GB_LR_3mm.pdf a 3mm acrylic sheet must be used.

5mm acrylic

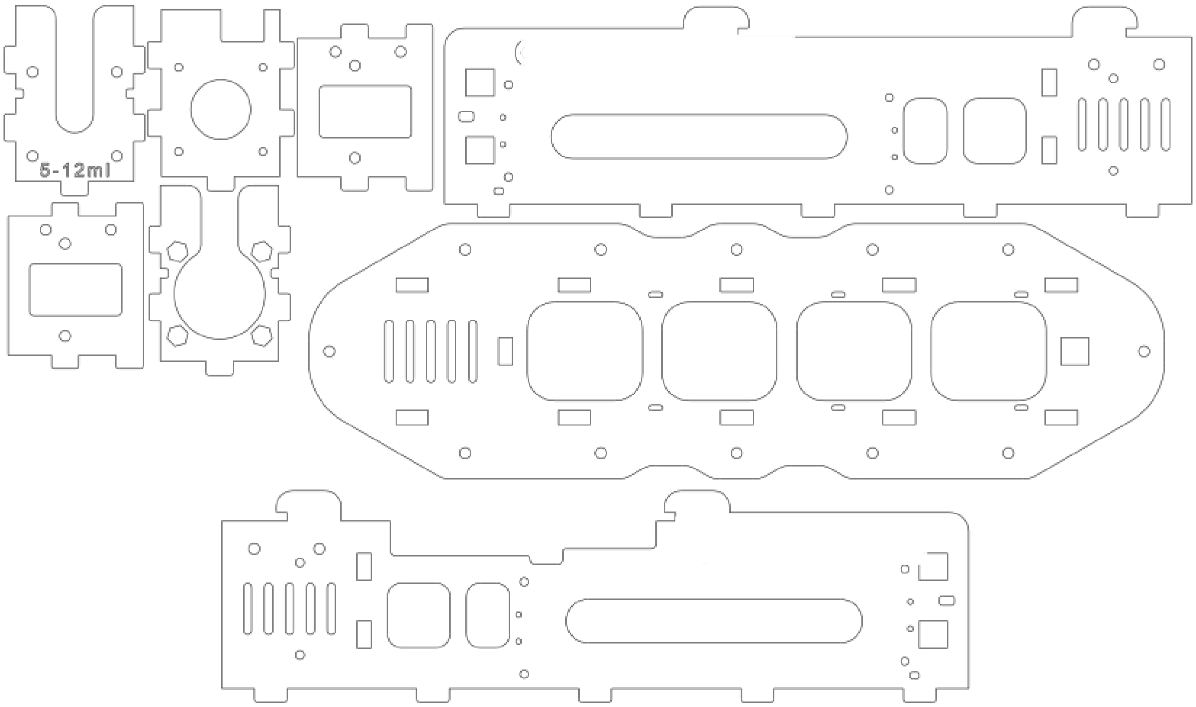


3 mm acrylic

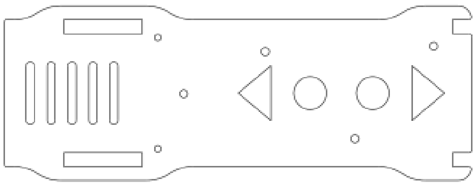

2. Assemble all the acrylic parts of the main body (1) (all the acrylic parts, except the cover) without gluing as depicted in the figures below (remove the acrylic protective cover prior to assembling).

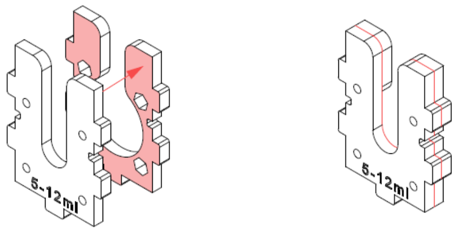


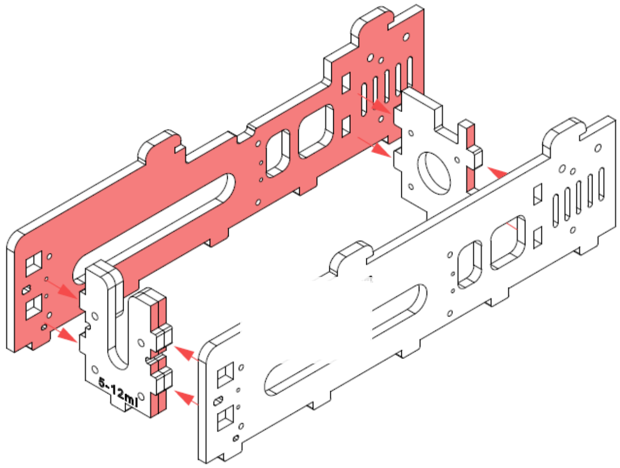


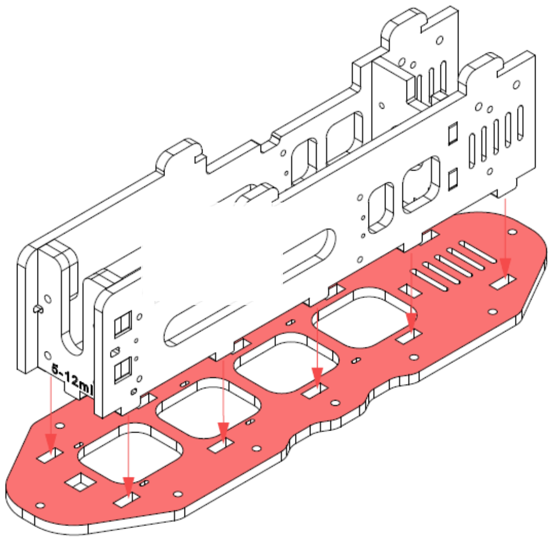

3. In order to ensure a more robust gluing and minimize the effects ot the acrylic tolerances, prior to gluing ensure that:
    a. Squeeze gently the side wall inwards and press the inside parts outwards

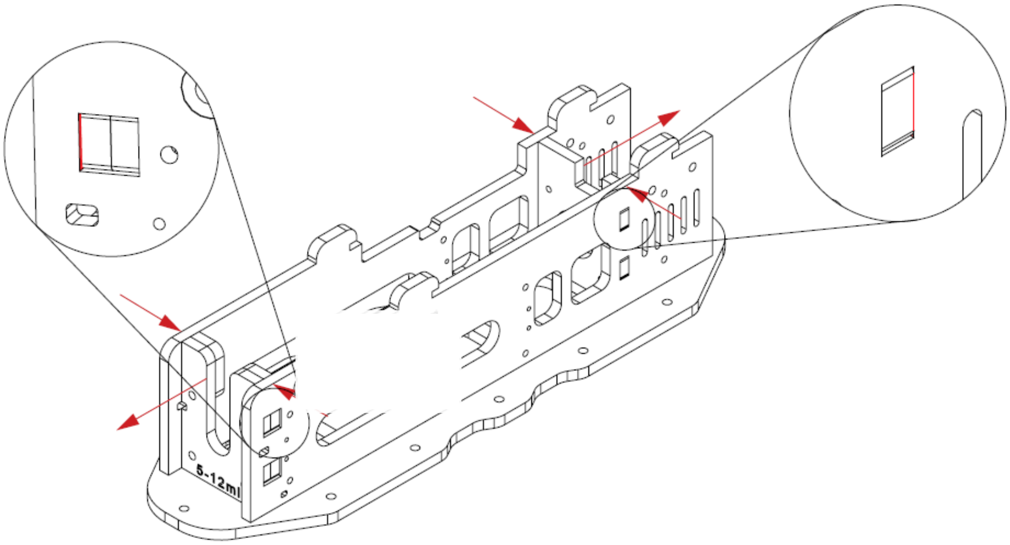


    b. Make sure that the main walls are parallel to each other. Check that the walls are in line with the markers of the bottom acrylic.

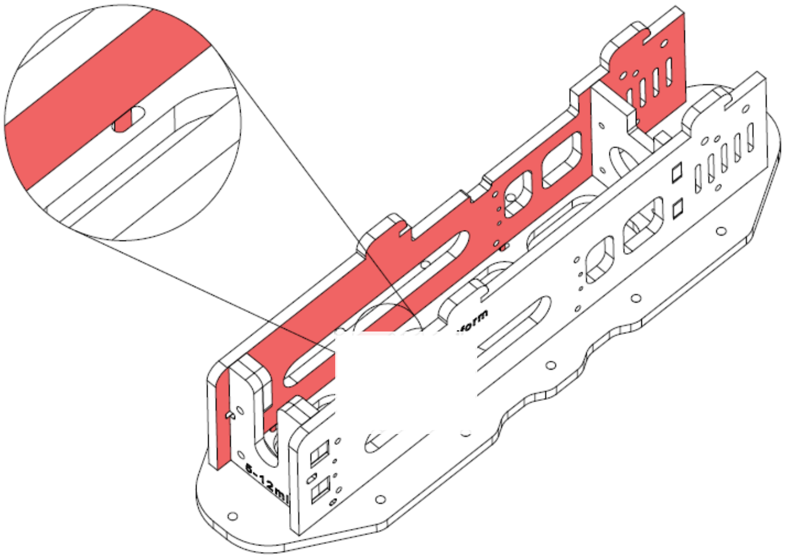

4. Using a brush, apply acrylic glue to the assembled structure.
   **Note:** It is recommended that the gluing should be done on top of a glass to avoid that any spills might damage the desk)
   a. Apply a thin layer of of glue on the back wall with one hand, while holding the assembled structure with the other hand;

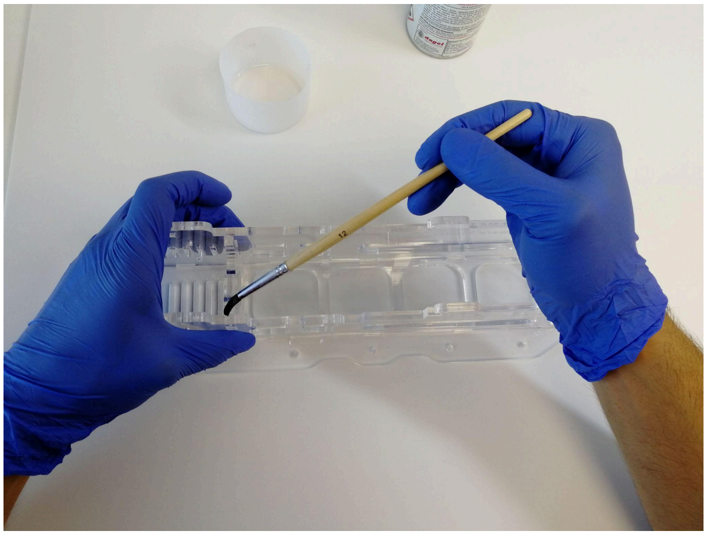

   b. Repeat the same procedure for the front wall with one hand, while holding the assembled structure with the other hand;

   c. Apply a thin layer of glue on the lateral walls while making sure that those walls are in line with the markers of the bottom acrylic, i.e., parallel to each other. You can slightly move the acrylic walls to make sure they are glued as parallel as possible.
   **Note:** You can use a ruler to help in this procedure

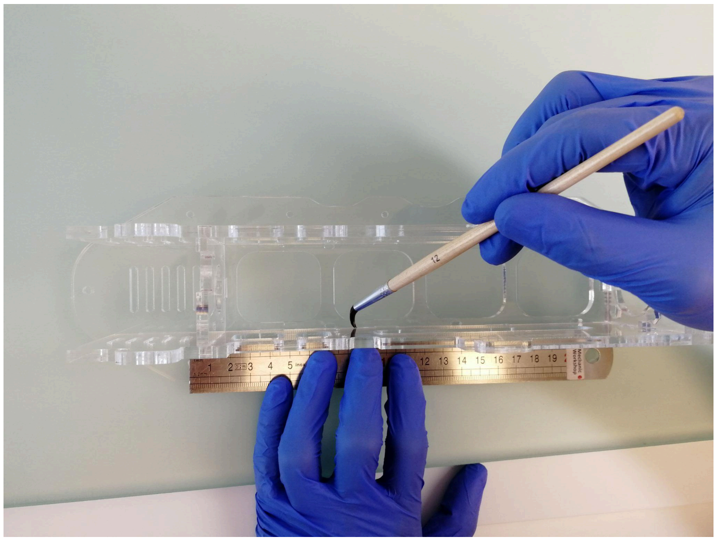

d. Apply glue to all of the structure edges to make sure the acrylic parts are bonded to each other. Turn the structure upside down and glue the bottom edges.

5. 3D print the STL files available in the repository, namely:

    - 1x 3D Printed Split Block Bottom (5)
    - 1x 3D Printed Split Block Top (6)
    - 1x 3D Printed Lock Tight Nut (7)
    - 1x 3D Printed Syringe Block Holder Top (15)
    - 2x 3D Printed Side Slider (20)
    - 1x 3D Printed Slider - non-Switch Side (22)
    - 1x 3D Printed Slide Block (24)
    - 2x 3D Printed End Switch Cover (27)
    - 2x 3D Printed End Switch Base (29)
    - 1x 3D Printed Syringe Block Holder Bottom (33)

6. Glue with super glue (Loctite 401 for example) the nuts to the printed parts. For each nut, use a correspondent screw to pull the nut towards the part in order to make sure it is properly attached to it

    a. Glue the the brass hex nut M5 (8) to part (7)

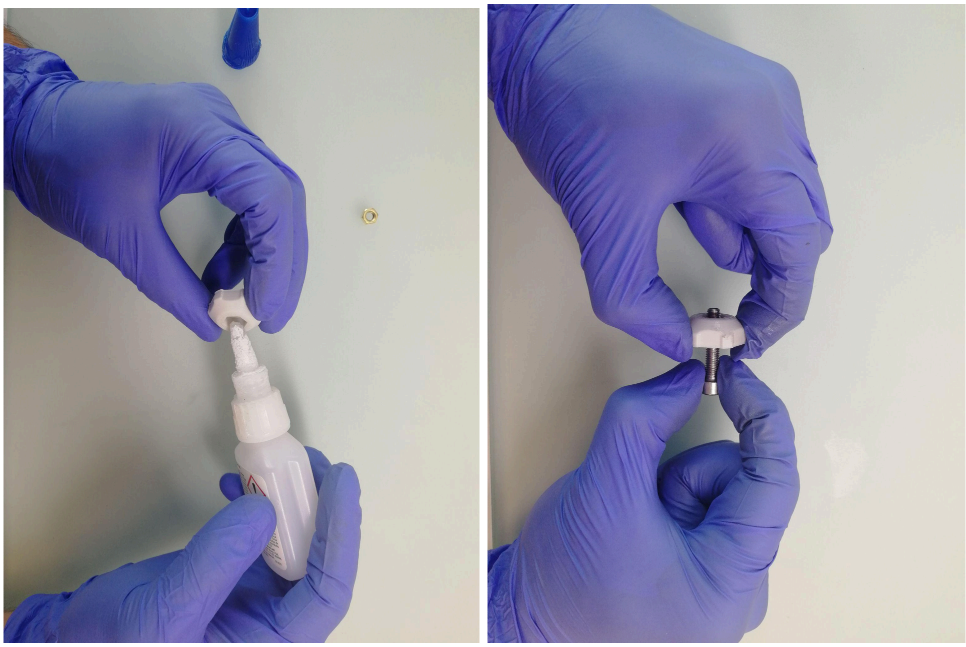

b. Glue a M4 square steel nut (10) to part (6)

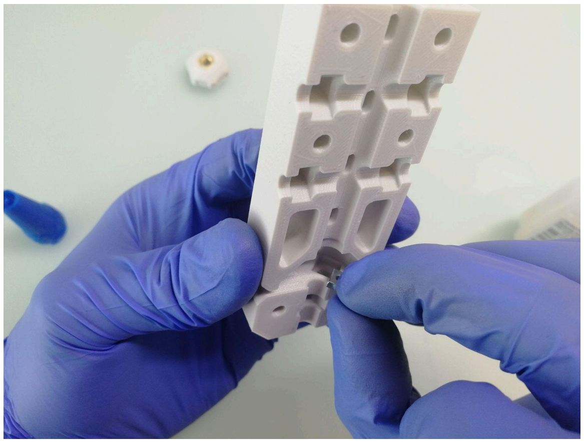

c. Glue a M4 square steel nut (10) to part (5)

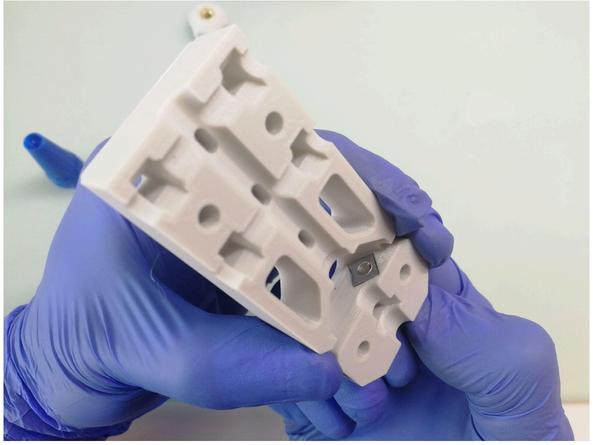

d. Glue 4x M4 hex nuts (12) to part (5)

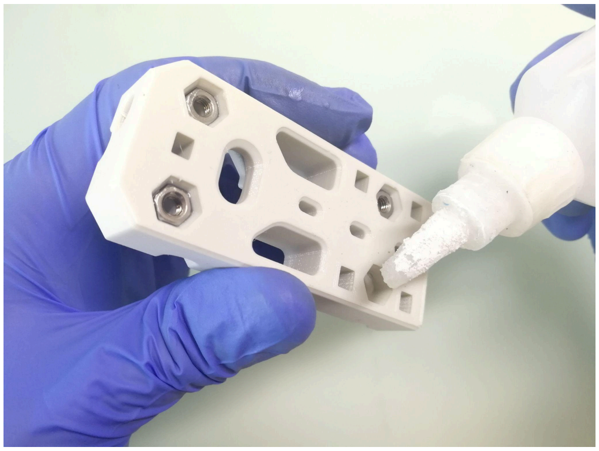

e. Glue 2x M4 hex nuts (12) and 2x M4x25mm hex screws (17) to part (33)

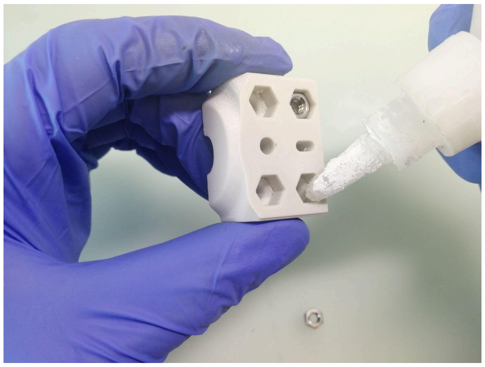
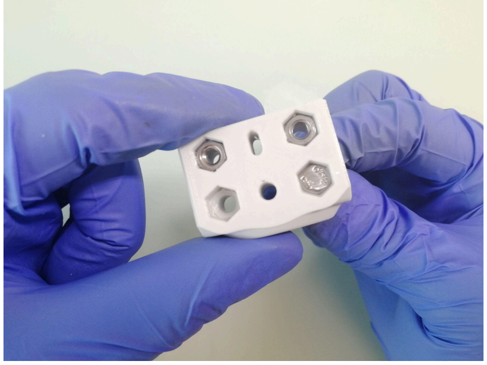

f. Glue 4x M4x25mm hex screw (17) to the main acrylic body. Place the 3D printed part while gluing the screws to align the screws

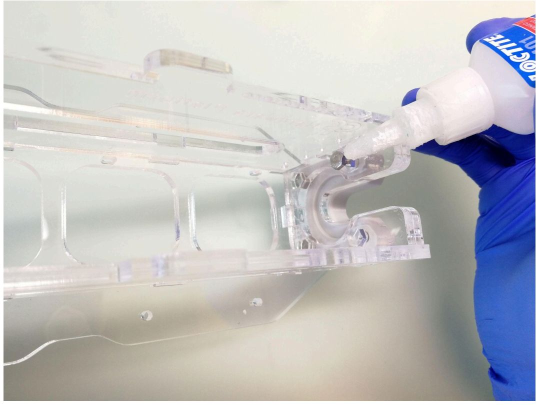

g. Glue 2x M2.5 hex nuts to part (29) for each one of the 3D switch base

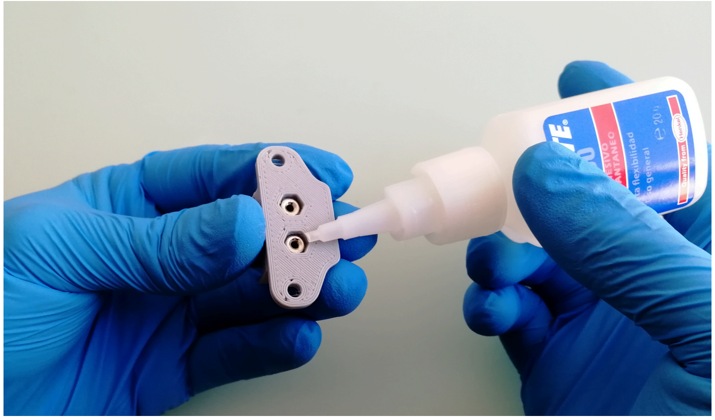

7. Insert the backlash nut and a brass nut (8) into the M5 rod (9)

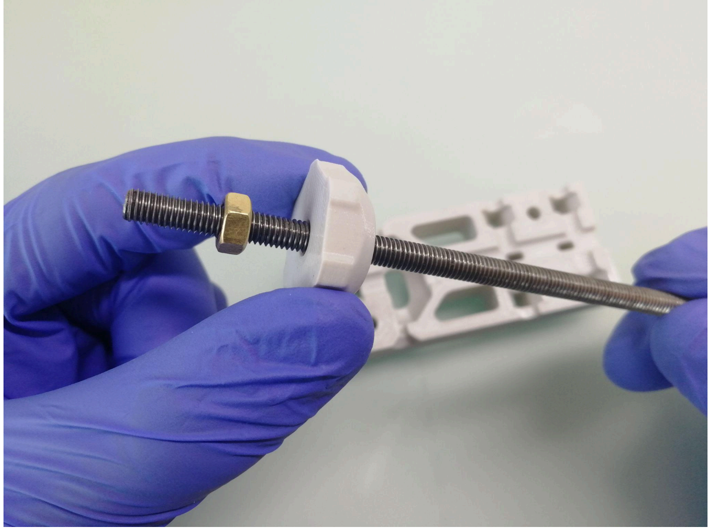

8. Place the rod with the nuts and place 4x M5 lock nuts (19). Before placing the top part, make sure that the backlash nut is loose, i.e, the rod rotates freely and the nut is not pressed against the printed part. Attach the top part and screw with the 4x M4x25mm hex socket cap screw (11)

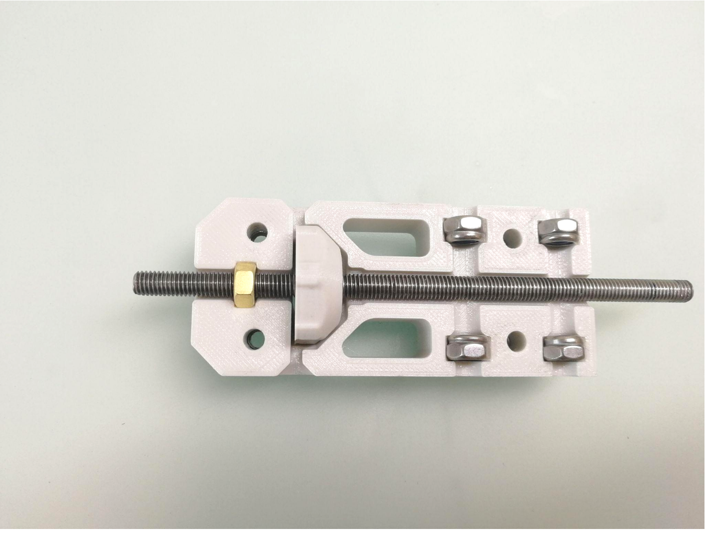

9. With a screwdriver inserted in the bottom hole, tighten the nut by pressing downwards until the rod cannot move completely and no backlash is felt.
   Attach the 2x M4x16mm hex socket cap screw (14) and tight the screw until you can feel that the rod is starting to rotate freely.

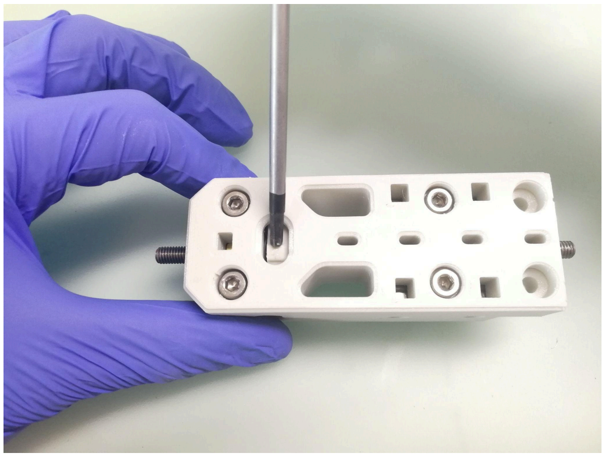
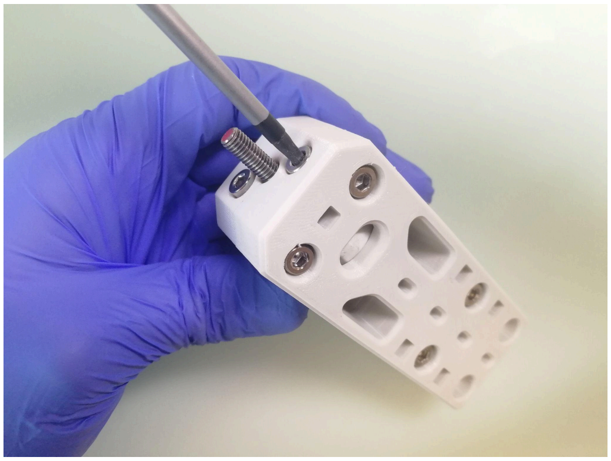

10. Screw and glue thumb nuts (16) on the edge of the M4 rods (25)

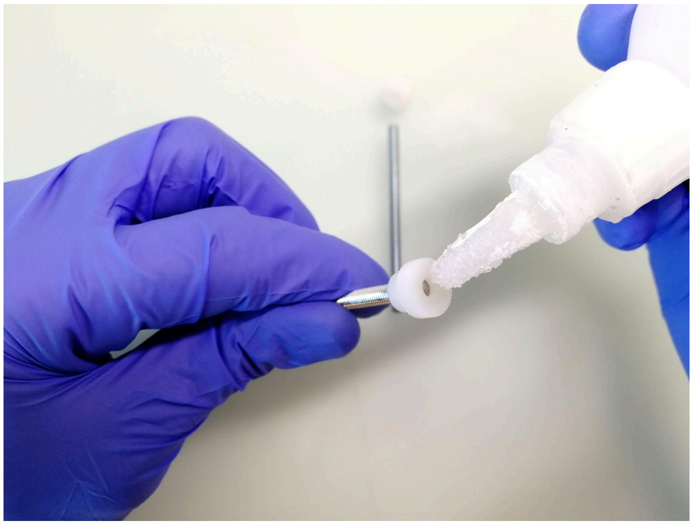

11. Insert the rods on the part (24) while placing a thumb nub (16) in the middle and on the opposite edge

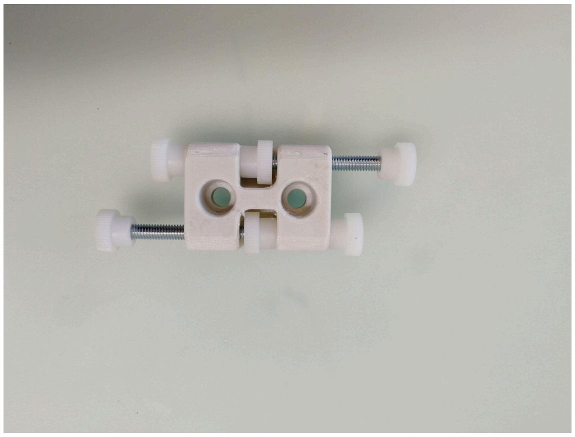

12. Attach the motor to the acrylic body using a 5/64  inches screwdriver to screw the 4x #4-40 screws (4)

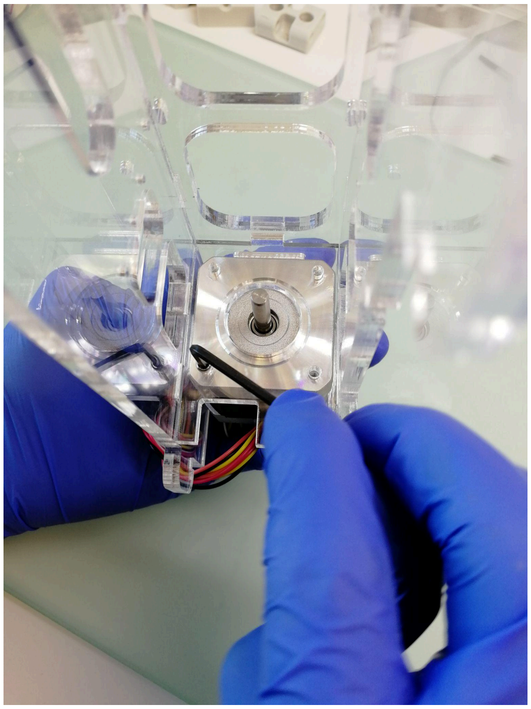

13. Using 2x M5x25mm hex socket screw (23), the parts (20) and (22) and the ball bearings (21), place them as depicted below

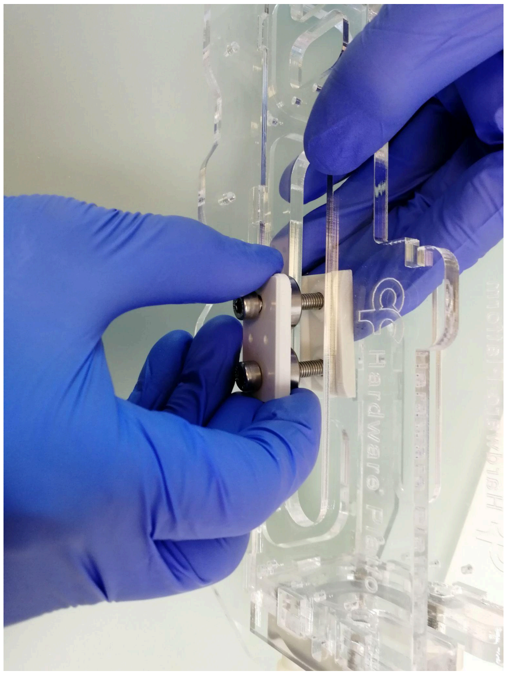

14. Add the main body and screw the M5 screws into the 3d part

15. Using 2x M5x25mm hex socket screw (23), the parts (25) and (20) and the ball bearings (21), place them as depicted below

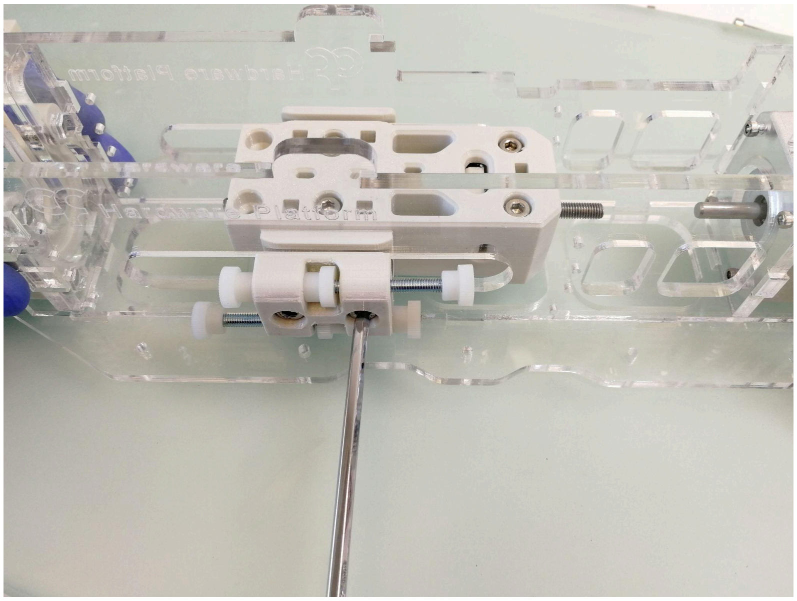

16. Add the coupler (13) to the motor shaft (3) and M5 rod (9). Important: The shaft and the rod inside the coupler must not be in contact with each other. A thread distance is recommended between them (0.8mm).

    a. First screw the coupler into the motor shaft so that the flat part of the shaft is aligned with one of the screws. Attach first that screw not very tight and then attach the second one also not very tight. Then repeat and screw both tightly.

    b. On the screws of the rod side, repeat the same process, making sure that the first screw to be screwed is the one that is aligned with whe first one that was screwed on the motor side.

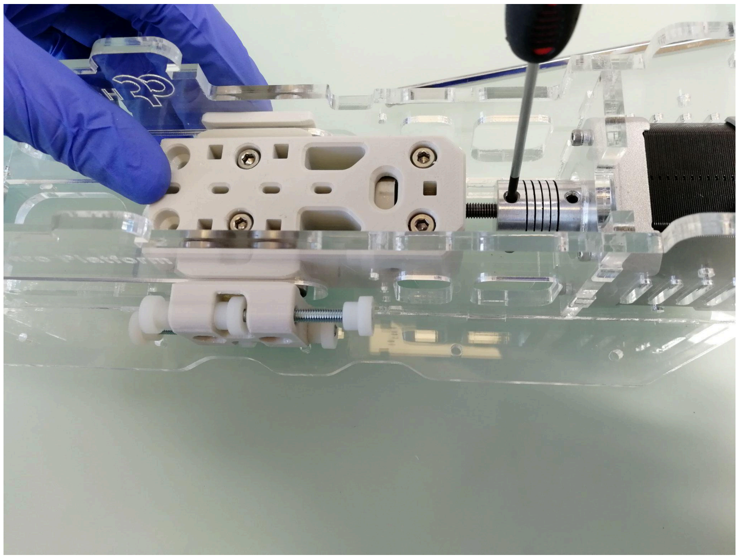

17. Solder two wires to each limit switch (28) that will connect to the controller board. The forward limit switch wire (in red) should have a length of approximately 37cm whereas the reverse limit switch wires (in black) should have a length of 18 cm.

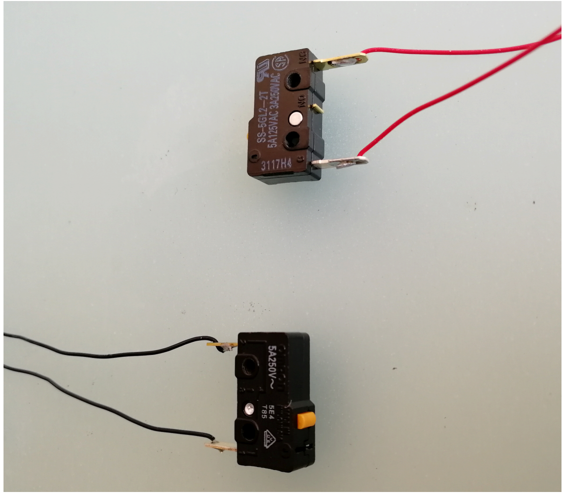

18. Place the switches as depicted in the picture below, with the front part of the switch aligned with the straight edge of part (29) and with the orange button closer to the top of (29) when this part is positioned vertically and facing the right side.

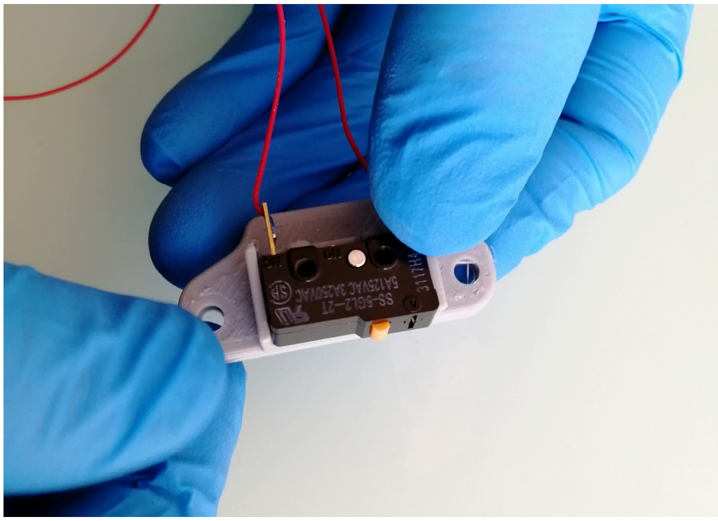

19. Add the 3D switch cover (27) and screw 2x M2.5 x 12mm Hex Socket Screw (26) to each switch limiter

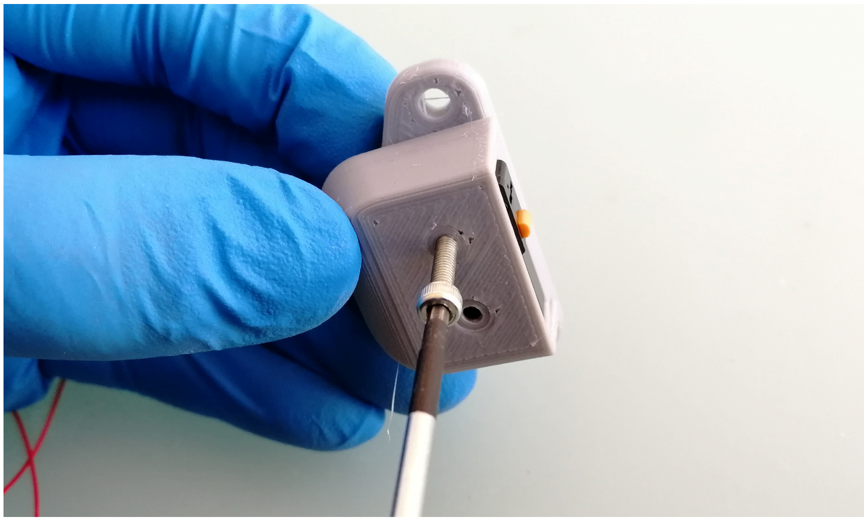

20. Use 2x No.4 x 13mm hex socket screw and 2x M3 lock nut for each switch, to fasten the switches into the acrylic main body

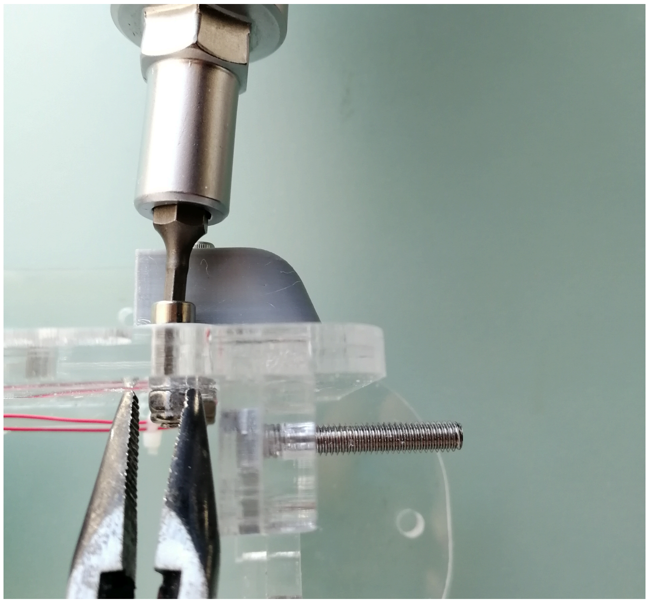

21. Using some nylon cable ties, secure the switch wires into the acrylic body in the locations suggested below

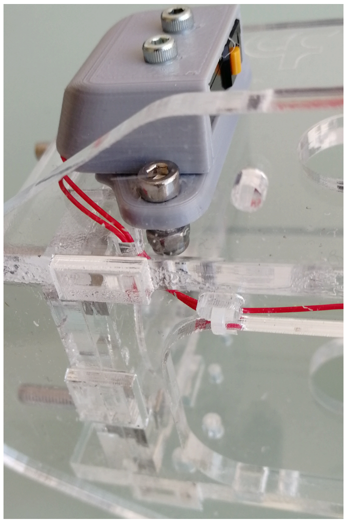

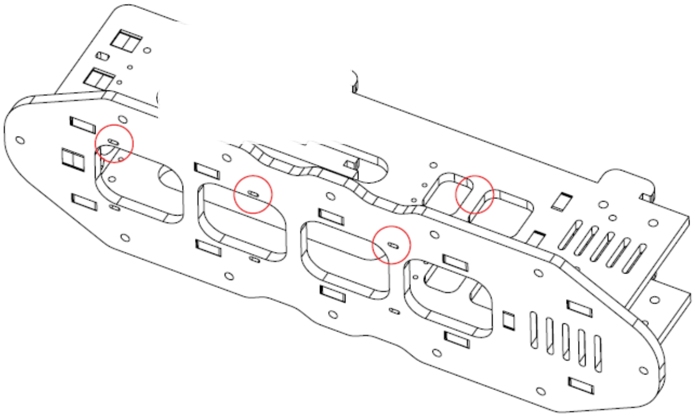

22. Attach the 4x M3 nylon screws (36) and 4x M3 nylon (35) to secure the Harp pump board into the acrylic part 2. Place one M3 nylon nut (35) between the acrylic part and the board.

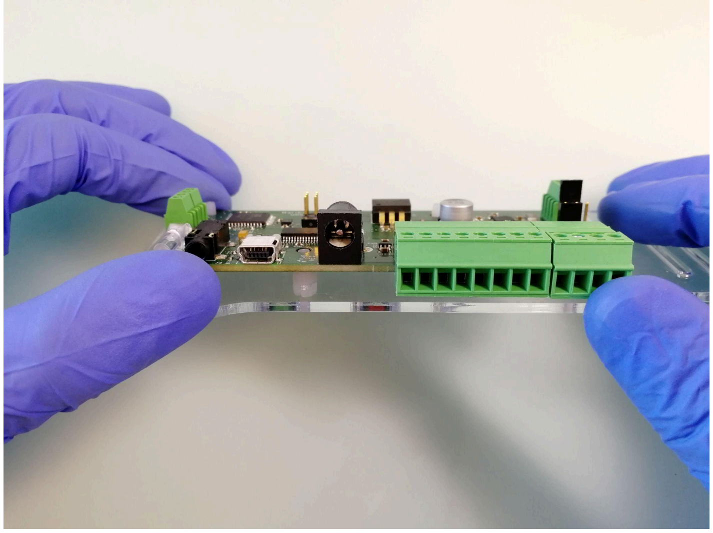

23. Attach the motor wires to the board in bipolar parallel connection, meaning that the wires will be connected in pairs: red white/yellow -> 1B; red/yellow white -> 1A, orange white/black -> 2A, orange / black white -> 2B.
Screw the switch wires, so that the red wires - forward limit switch - are connected to SW_F connector and black wires - reverse limit switch - are connected to SW_R connector.

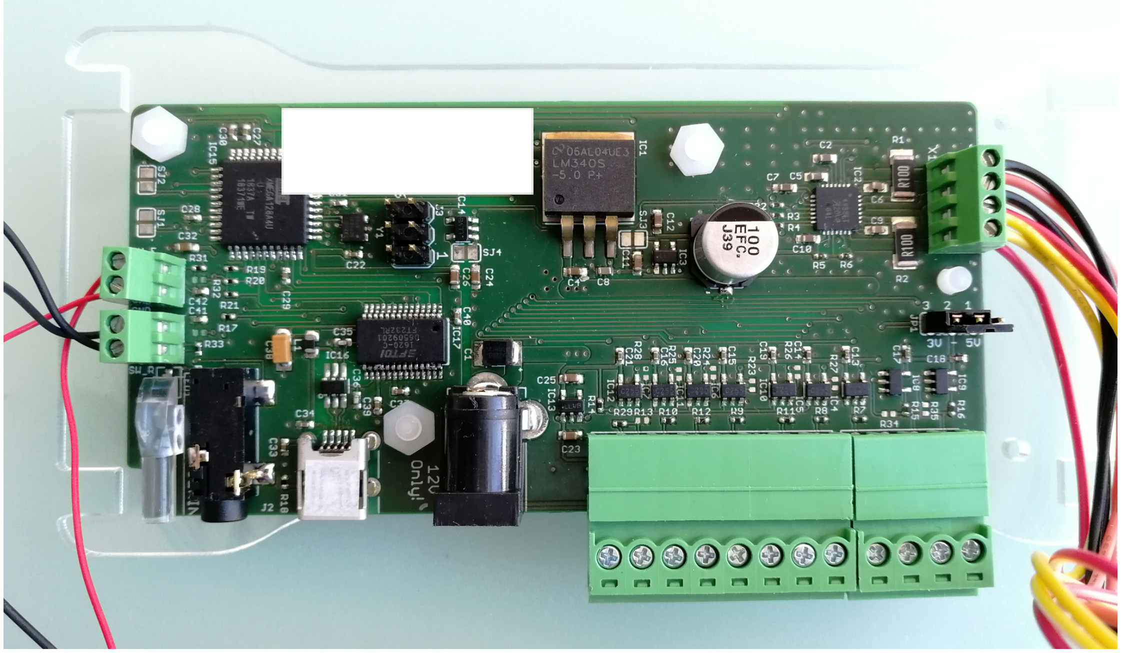

24. Attach part (18) with the thumb nuts (16) and attach the top lid (2) with the controller to the acrylic body.

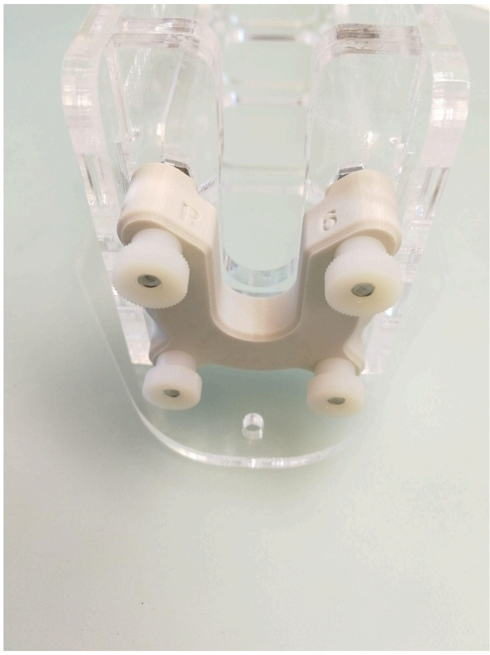

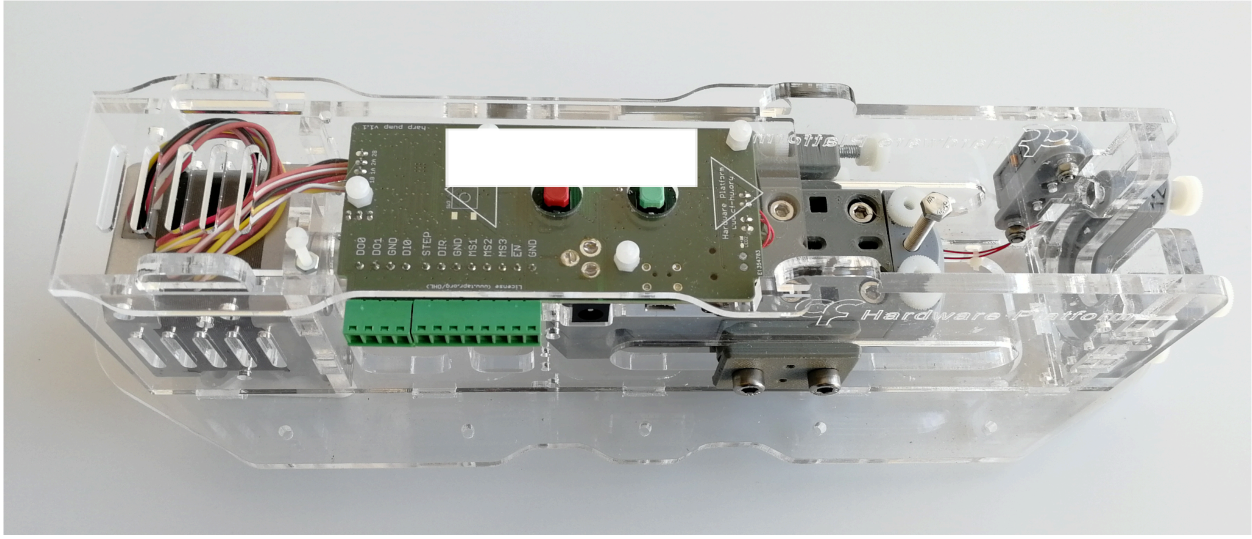

25. Add the 4x M4 stud mounts (37) and respective M4 lock nuts (38) to the base of the syringe pump

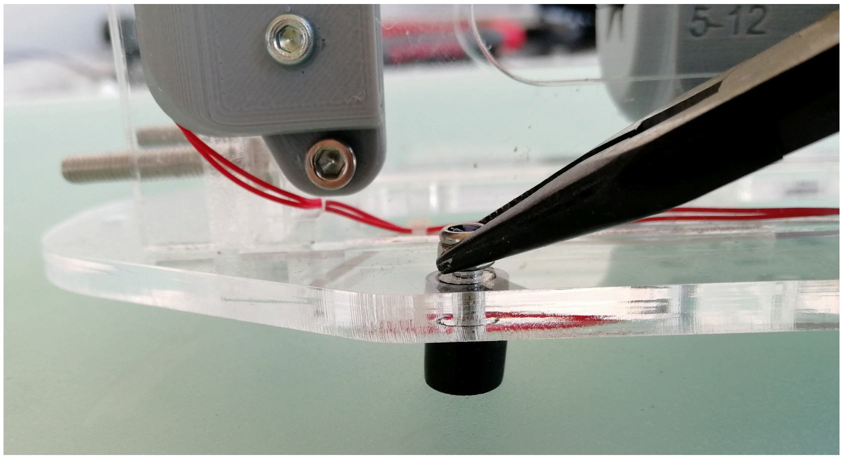

## Assembled Syringe Pump

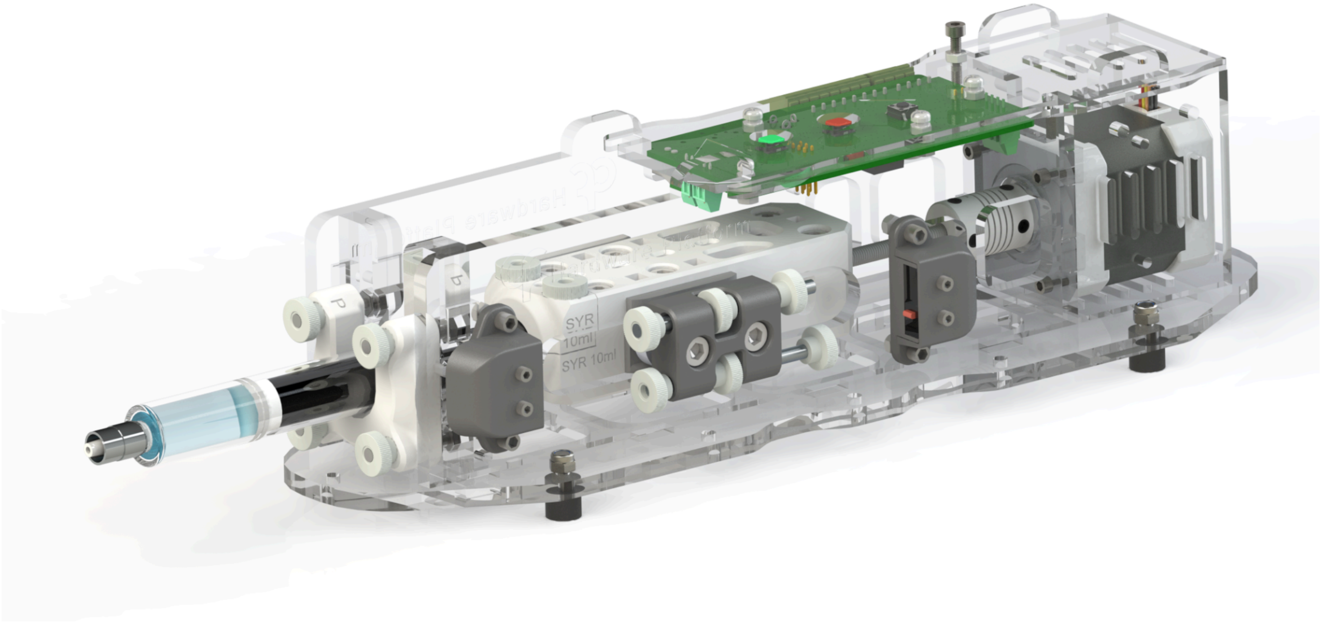

Supplement: Data 1 — Resource files for the interface software, firmware, mechanical designs and assembly. Download Data 1, ZIP file. [file eneuro-12-ENEURO.0024-25.2025-s004.zip › Extended Data 1/Hardware/CAD/Assembly_Instructions_blind.pdf]
