# Supplementary material for: A Flexible Fluid Delivery System for Rodent Behavior Experiments
Source: eNeuro. 2025 Jul 24;12(7):ENEURO.0024-25.2025. doi: 10.1523/ENEURO.0024-25.2025 (PMC12320920; doi:10.1523/ENEURO.0024-25.2025)
Supplement: Data 1 — Resource files for the interface software, firmware, mechanical designs and assembly. Download Data 1, ZIP file. [file eneuro-12-ENEURO.0024-25.2025-s004.zip › Extended Data 1/Hardware/CAD/Syringe_Pump_exploded_descritive_view_blind.pdf]

Syringe Pump Exploded View

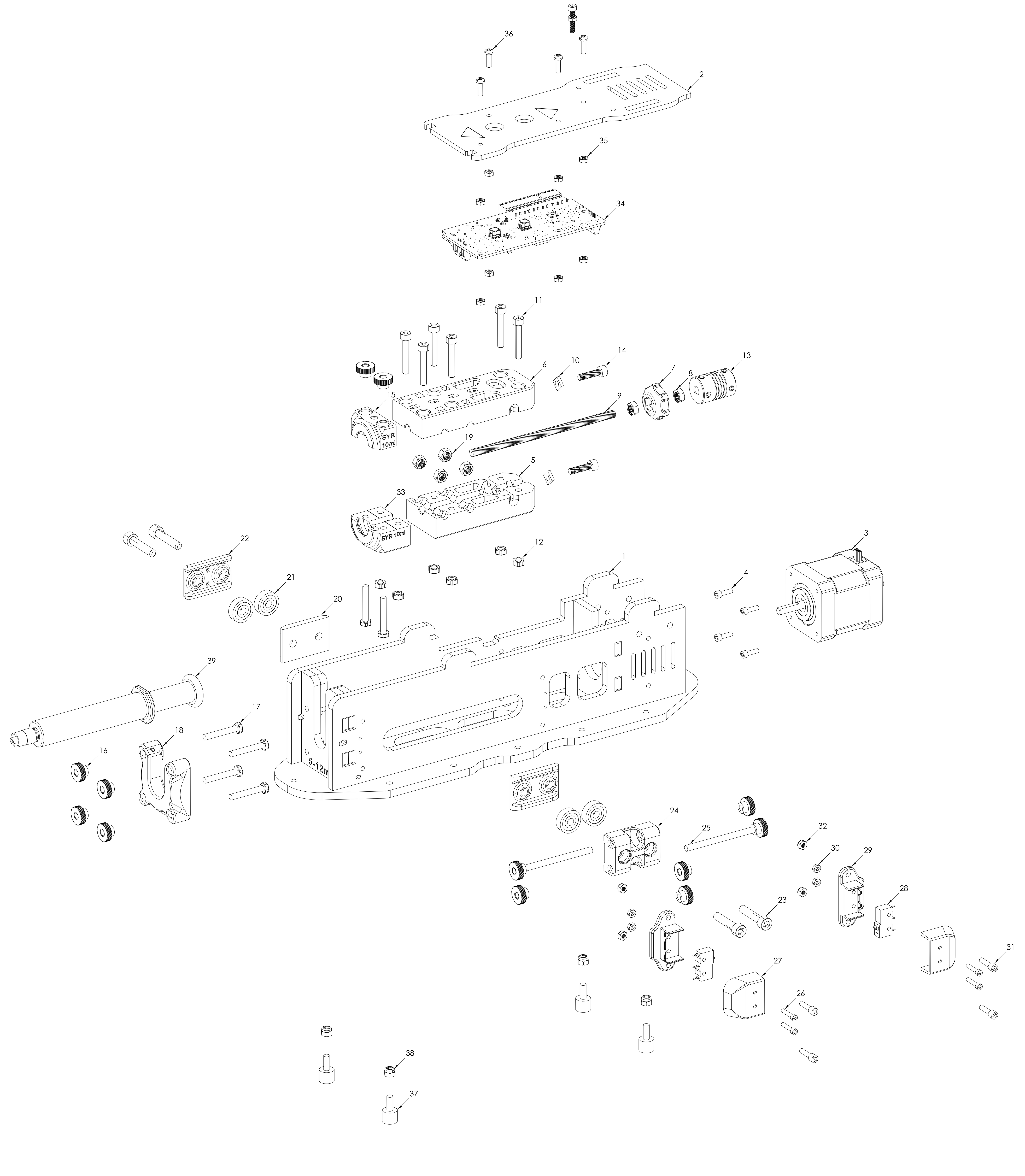

1. Acrylic body
2. Acrylic lid
3. Nema 17 stepper motor
4. #4-40 x 3/8" Hex Socket Screw (4X)
5. 3D Printed Split Block Bottom
6. 3D Printed Split Block Top
7. 3D Printed Lock Tight Nut
8. M5 Brass Hex Nut
9. Threaded Rod M5
10. M4 7mm Steel Square Nut (2X)
11. M4 x 25mm Hex Socket Screw (6X)
12. M4 Hex Nut (6X)
13. M5 to M5 Coupler
14. M4 x 16mm Hex Socket Screw (2X)
15. 3D Printed Syringe Block Holder Top
16. M4 Thumb Nut (12X)
17. M4 x 25mm Hex Bolt (6X)
18. 3D Printed Syringe Locker Plate
19. M5 Nylon Insert Lock Nut (4X)
20. 3D Printed Side Slider (2X)
21. Ball Bearing 5mm - 16mm (4X)
22. 3D Printed Slider - non-Switch Side
23. M5 x 25mm Hex Socket Screw (4X)
24. 3D Printed Slide Block
25. Threaded Rod M4 (2X)
26. M2.5 x 12mm Hex Socket Screw (5X)
27. 3D Printed End Switch Cover (2X)
28. End Switch (2X)
29. 3D Printed End Switch Base (2X)
30. M2.5 Hex Nut (5X)
31. No.4 x 13mm Hex Socket Screw (4X)
32. M3 Lock Nut (4X)
33. 3D Printed Syringe Block Holder Bottom
34. PCB Harp Board
35. M3 x16mm Nylon Nut (8X)
36. M3 x16mm Nylon Screw (4X)
37. M4 Stud Mount (4X)
38. M4 Lock Nut (4X)
39. Syringe
