# Supplementary figures and images for: A Flexible Fluid Delivery System for Rodent Behavior Experiments
Source: eNeuro. 2025 Jul 24;12(7):ENEURO.0024-25.2025. doi: 10.1523/ENEURO.0024-25.2025 (PMC12320920; doi:10.1523/ENEURO.0024-25.2025)

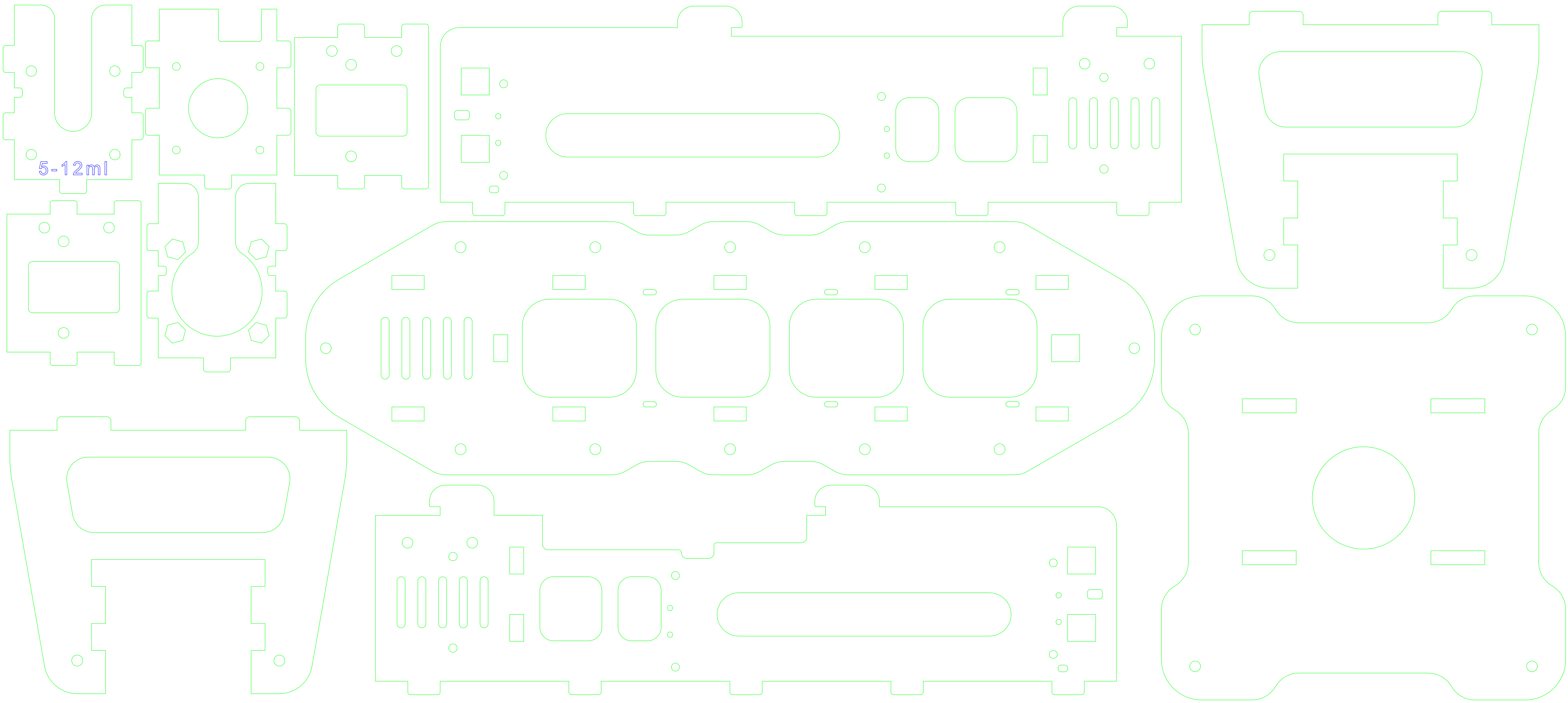

5-12ml

Supplement: Data 1 — Resource files for the interface software, firmware, mechanical designs and assembly. Download Data 1, ZIP file. [file eneuro-12-ENEURO.0024-25.2025-s004.zip › Extended Data 1/Hardware/CAD/1_Acrylic_Parts_Syringe_Body_5mm_v1_blind.pdf]

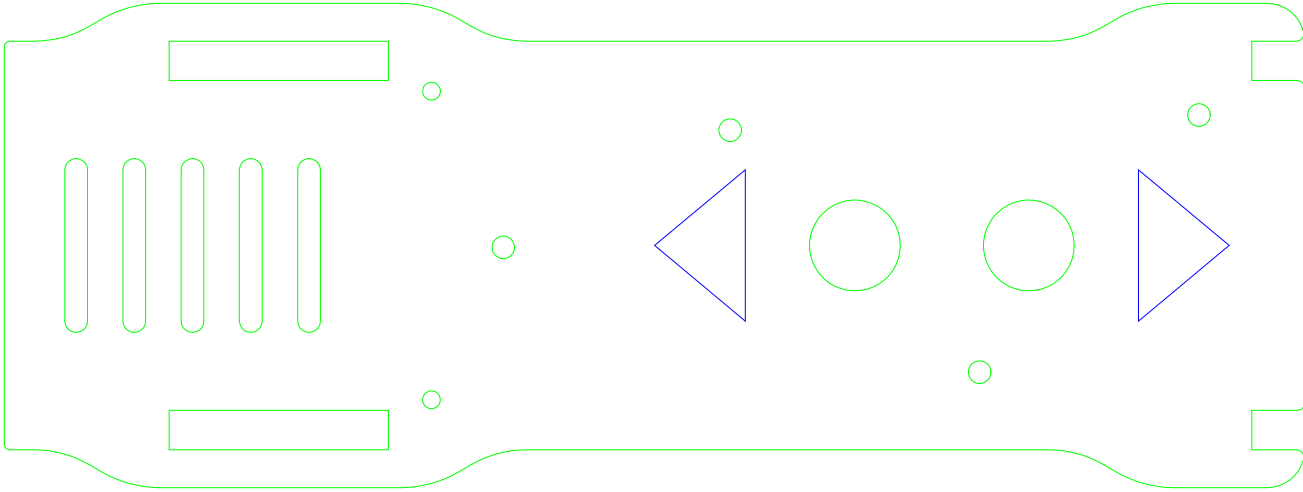

Supplement: Data 1 — Resource files for the interface software, firmware, mechanical designs and assembly. Download Data 1, ZIP file. [file eneuro-12-ENEURO.0024-25.2025-s004.zip › Extended Data 1/Hardware/CAD/2_Acrylic_Parts_Syringe_Top_3mm_v1_blind.pdf]
